# Supplementary material for: Visualization and standardized quantification of surface charge density for triboelectric materials
Source: Nat Commun. 2024 Jul 17;15:6004. doi: 10.1038/s41467-024-49660-9 (PMC11255240; doi:10.1038/s41467-024-49660-9)
Supplement: Supplementary file 1 — Supporting Information [file 41467_2024_49660_MOESM1_ESM.pdf]

Supplementary Information

Visualization and Standardized Quantification of Surface Charge Density for Triboelectric Materials

Yi Li [1, #], Yi Luo [2, #], Song Xiao [1, #], Cheng Zhang [2], Cheng Pan [1], Fuping Zeng [1], Zhaolun Cui [3], Bangdou Huang [2], Ju Tang [1], Tao Shao [2,*], Xiaoxing Zhang [4, *], Jiaqing Xiong [5,*], Zhong Lin Wang [6, 7 *]

[1] State Key Laboratory of Power Grid Environmental Protection, School of Electrical Engineering and Automation, Wuhan University, Wuhan, Hubei 430072, People's Republic of China

[2] Beijing International S&T Cooperation Base for Plasma Science and Energy Conversion, Institute of Electrical Engineering, Chinese Academy of Sciences, Beijing 100190, People's Republic of China.

[3] School of Electric Power Engineering, South China University of Technology, Guangzhou, 510641, People's Republic of China

[4] Key Laboratory for High-Efficiency Utilization of Solar Energy and Operation Control of Energy Storage System, School of Electrical and Electronic Engineering, Hubei University of Technology, Wuhan 430068, People's Republic of China

[5] Innovation Center for Textile Science and Technology, Donghua University, Shanghai, 201620, People's Republic of China

[6] Beijing Institute of Nanoenergy and Nanosystems, Chinese Academy of Sciences, Beijing 100083, People's Republic of China

[7] School of Materials Science and Engineering, Georgia Institute of Technology, Atlanta, Georgia, 30332-0245, United States

[*] Corresponding authors: Zhong Lin Wang, wangzhonglin@binn.cas.cn, Jiaqing Xiong jqxiong@dhu.edu.cn, Xiaoxing Zhang zhangxx@hbut.edu.cn, Tao Shao, st@mail.iee.ac.cn

[#] The authors contributed equally to this work.

This PDF file includes

Supplementary Notes. 1-9
Supplementary Figs. 1-64
Supplementary References

Supplementary Notes

Supplementary Note 1 | Relationship Between Surface Potential and Surface Charge

The surface potential and charge density ($\boldsymbol{\sigma}$) within each element can be expressed as[1],

$$\begin{bmatrix} \varphi_1 \\ \vdots \\ \varphi_i \\ \vdots \\ \varphi_n \end{bmatrix}_{n\times 1} = \begin{bmatrix} h_{11} & \cdots & h_{1j} & \cdots & h_{1n} \\ \vdots & \ddots & \vdots & \ddots & \vdots \\ h_{i1} & \cdots & h_{ij} & \cdots & h_{in} \\ \vdots & \ddots & \vdots & \ddots & \vdots \\ h_{n1} & \cdots & h_{nj} & \cdots & h_{nn} \end{bmatrix}_{n\times n} \begin{bmatrix} \sigma_1 \\ \vdots \\ \sigma_i \\ \vdots \\ \sigma_n \end{bmatrix}_{n\times 1} \quad (1)$$

where φ_i and σ_i is the surface potential and charge density at point i, respectively.

If only the ith row of the charge matrix $\boldsymbol{\sigma}$ is not zero, then equation (1) can be transformed into:

$$\varphi_i = \mathbf{H}_{n\times n}^{j}\sigma_i \quad (2)$$

where $\mathbf{H}_{n\times n}^{j}$ is the jth column of the matrix $\mathbf{H}_{n\times n}$. Therefore, it is only necessary to set σ_i=1 to obtain the data of the jth column of $\mathbf{H}_{n\times n}$. It can be seen that the matrix $\mathbf{H}_{n\times n}$ is independent of the surface charge distribution but only related to the structure of the measurement system. Thus, take turns setting the charge density of each element to 1 C m^{-2}, and record the surface potential of all elements after each computation. After repeating n times, and performing numerical transformation according to the properties of the circulant matrix, the complete transfer function matrix $\mathbf{H}_{n\times n}$ can be obtained. Then, the surface charge distribution can be obtained only by inverting $\mathbf{H}$:

$$\boldsymbol{\sigma}_{n\times 1}=\mathbf{H}_{n\times n}^{-1}\boldsymbol{\varphi}_{n\times 1} \quad (3)$$

Obviously, the dimension of the matrix $\mathbf{H}$ is closely related to the number of measurement points n. Since the charge simulation method treats the voltage and electric field in each element as uniformly distributed, it will cause larger measurement errors when the number of measurement points is small. Therefore, a larger number of measurement points is often selected to ensure measurement accuracy. Accordingly, the sensitivity and stability of the matrix for numerical calculation also need to be evaluated for large linear systems. If a linear system is ill-conditioned, even a slight disturbance can cause dramatic changes in the equation solution, and

the reliability of the results should be re-evaluated.

The condition number is used to measure the sensitivity of the output of a matrix multiplication or inversion to input errors[2]. The condition number of the matrix **H** is defined as:

$$\mathrm{cond}(\mathbf{H}) = \|\mathbf{H}\| \cdot \|\mathbf{H}^{-1}\| \tag{4}$$

where $\|\cdot\|$ is the Euclidean norm. The greater the condition number cond(**H**) is, the larger the error caused by the small disturbance and the lower the computation accuracy. It is generally believed that the matrix **H** is an orthogonal matrix and has the best numerical stability when the condition number is equal to 1. However, the shift-invariant system is not satisfied. For the measuring system in this paper, the condition number of the transfer function matrix **H** is cond(**H**)=13, which is much larger than 1. It can be considered that the matrix **H** is an ill-conditioned matrix, and (1) can be regarded as a linear discrete ill-posed problem.

Supplementary Note 2 | Flexible Golub-kahan (FGK) Transform

Formally, the FGK is closely related to the inexact Lanczos bi-diagonalization (LBD) [3]. For the surface inversion problem, the FGK generates a set of orthonormal basis $\mathbf{Z}_k = \left[I_1^{-1} v_1, I_2^{-1} v_2, \cdots, I_k^{-1} v_k \right] \in \mathbb{R}^{n \times k}$ and computes the estimated surface charge density by solving Eq. $\min\{\|\mathbf{H}\boldsymbol{\sigma} - \boldsymbol{\varphi}\|^2 + \lambda^2 \mathbf{I} \|\boldsymbol{\sigma}\|^2\}$ in the low-dimension projected subspace [4]. Then the iteration process at k^{th} iteration is:

$$\mathbf{H}\mathbf{Z}_k = \mathbf{W}_{k+1}\mathbf{D}_k \tag{5}$$

$$\mathbf{H}^{\mathrm{T}}\mathbf{W}_{k+1} = \mathbf{V}_{k+1}\mathbf{T}_{k+1} \tag{6}$$

where $\mathbf{D}_k$ is an upper Hessenberg matrix with dimension of $(k+1)\times k$, $\mathbf{T}_{k+1}$is an upper triangular matrix with a dimension of $(k+1)\times(k+1)$, the first column of $\mathbf{W}_k$ is $\varphi / \|\varphi\|$, and the initial solution $\boldsymbol{\sigma}_0=0$. The FGK process is shown in Algorithm 1.

Algorithm 1 Flexible Golub-kahan (FGK) decomposition

Initialize $w_1=\boldsymbol{\varphi}/\beta$, where $\beta=\|\boldsymbol{\varphi}\|$

for $j=1, \ldots, k$ do

  Compute $\mathbf{u}=\mathbf{H}^{\mathrm{T}}\mathbf{w}_j$, $t_{ij}=\mathbf{u}^{\mathrm{T}}\mathbf{v}_i$ for $i=1, 2, \ldots, j-1$

  Set $\mathbf{u} = \mathbf{u} - \sum_{i=1}^{j-1} t_{ij}\mathbf{v}_i$, compute $t_{jj}=\|\mathbf{u}\|$ and take $\mathbf{v}_j=\mathbf{u}/t_{jj}$

  Compute $\mathbf{z}_j = \mathbf{I}_j^{-1}\mathbf{v}_j$ and $\mathbf{u}=\mathbf{H}\mathbf{z}_j$

  $d_{ij}= \mathbf{u}^{\mathrm{T}}\mathbf{w}_i$ for $i=1, \ldots, j$ and set $\mathbf{u} = \mathbf{u} - \sum_{i=1}^{j} d_{ij}\mathbf{v}_i$

  Compute $d_{j+1,j}=\|\mathbf{u}\|$ and take $\mathbf{w}_{j+1}=\mathbf{u}/d_{j+1,j}$

end

Supplementary Note 3 | From GCV to WGCV method

The standard Generalized Cross Validation (GCV) method is inspired by cross-validation that divides the initial data into two parts. One is used to calculate the approximate solution, and the other is used to verify the approximate solution[5]. After sequentially removing each data in the measurement matrix **φ**, for the standard Tikhonov regularization problem ($\min\{\|\mathbf{H\sigma}-\boldsymbol{\varphi}\|^2+\lambda^2\mathbf{I}\|\boldsymbol{\sigma}\|^2\}$), the following standard GCV function is used to find the regularization parameter that minimizes the prediction error.

$$G(\lambda)=\frac{n\left\|(\mathbf{I}-\mathbf{HH}_{\lambda}^{\dagger})\boldsymbol{\varphi}\right\|_2}{(trace(\mathbf{I}-\mathbf{HH}_{\lambda}^{\dagger}))^2} \tag{7}$$

where $trace(\cdot)$ represents the trace of the matrix, and $\boldsymbol{H}_{\lambda}^{\dagger}$ represents the pseudo-inverse of the matrix $[\mathbf{H}, \lambda\mathbf{I}]^{\mathrm{T}}$.

The standard GCV is an accurate regularization parameter selection method when the noise is a Gaussian signal for the standard Tikhonov regularization. However, the GCV function is too flat near the minimum point for some specific problems, which can easily cause overestimation. When using the GCV method to select the regularization parameters of the projected regularization problem, the solution error is not well stabilized as the iteration proceeds. To this end, a weight coefficient ω is introduced into the standard GCV function [6]. Accordingly, the Weighted GCV (WGCV) function can be expressed as:

$$G(\omega,\lambda)=\frac{n\left\|(\mathbf{I}-\mathbf{HH}_{\lambda}^{\dagger})\boldsymbol{\varphi}\right\|_2}{(trace(\mathbf{I}-\omega\mathbf{HH}_{\lambda}^{\dagger}))^2} \tag{8}$$

When ω=1, Eq. (8) degenerates into standard GCV; if ω>1, an over-smoothed solution is obtained; if ω<1, the obtained solution is under-smoothed. Currently, the weight coefficient ω is mostly selected by experiment. Specifically, by comparing the solution $\boldsymbol{\sigma}_k$ at the kth iteration under different ω with a given exact solution $\boldsymbol{\sigma}_{\mathrm{true}}$, the optimal weight parameter can be determined when the relative error $\|\boldsymbol{\sigma}_k-\boldsymbol{\sigma}_{true}\|_2/\|\boldsymbol{\sigma}_{true}\|_2$ is the smallest. However, in practice, due to the noise information and randomness in the measurement data, the weight coefficient selected by the experiment is not suitable for all cases, and the method is also affected by subjective factors. Therefore, an adaptive WGCV (A-WGCV) method is adopted to automatically select the

regularization parameters for each iteration of the projected least squares problem.

Supplementary Note 4 | Inversion Algorithm Evaluation

The SNR and PMSE are introduced to evaluate the proposed algorithm's inversion accuracy objectively [7]. The calculation methods of SNR and PMSE are as follows:

$$\mathrm{SNR} = -10\log_{10}\frac{\sum_{i=1}^{n}\{\tilde{\sigma}_i - \sigma_i\}^2}{nA^2} \tag{9}$$

$$\sqrt{\mathrm{PMSE}} = \sqrt{\frac{\sum_{i=1}^{n}\{\tilde{\sigma}_i - \sigma_i\}^2}{nA^2}} \tag{10}$$

where σ_i is the element in the real surface charge $\boldsymbol{\sigma}_\mathrm{true}$, $\tilde{\sigma}_i$ is the element in the estimated surface charge density $\tilde{\boldsymbol{\sigma}}$, and A is the maximum value of σ_i. The higher the SNR and the smaller the PMSE, the higher the image quality.

The relative error is also introduced to evaluate the gap between the estimated and real surface charge, which is defined as:

$$e = \frac{\left\|\boldsymbol{\sigma}_{true} - \tilde{\boldsymbol{\sigma}}\right\|_2}{\left\|\boldsymbol{\sigma}_{true}\right\|_2} \tag{11}$$

Supplementary Note 5 | Dust figure method

Once the blower disperses the dust particles into the air, they descend naturally due to gravity and are attracted to the surface charge, which has a polarity opposite to that of the dust. Here, the blue carbon powder with an average particle size of 5μm and a single positive polarity is used. Consequently, the powder is attracted to the negatively charged region on the sample, forming a dust figure that accurately outlines the charge distribution.

Supplementary Note 6 | The influence of probe-to-sample distance

The change in probe-to-surface distance will only impact the precision of surface potential measurement and have any influence on the universality of the proposed charge visualization and standardized quantification method. The Kelvin electrostatic probe was employed to scan the sample's surface potential distribution. The sample surface was divided into grids with *n* elements. Then, the surface potential and surface charge distribution were expressed in the following matrix form, where $\mathbf{H}_{n \times n}$ is defined as the transfer function matrix for surface charge inversion calculation. It is evident that the **H** matrix is independent of the distance between the electrostatic probe and the sample. The only restriction on our suggested charge visualization and standardized quantification method is related to the surface potential input matrix (size and structure). There are no demands for the device or potential measurement parameter settings.

Moreover, the measurement accuracy of surface potential is determined by the operating principle of the electrostatic probe, and it can be affected by variations in the probe-to-surface distance. In this work, the electrostatic potentiometer (TREK 341B) is used, which has a maximum measurement error of ± 0.1% and a measurement range of -19.99 kV to +19.99 kV. According to the equipment handbook, the suggested distance between the probe and the sample is 3 mm ± 1 mm (2-4 mm). Additionally, we examined the surface potential measurement results at various probe-to-sample distances (1–15 mm) and discovered that the average surface potential (-1570 ~ -1572V) was observed at a distance of 1-4 mm. The surface potential measurement findings will gradually be underestimated as the distance increases to higher than 5 mm. In particular, the test results will be badly skewed as the distance exceeds 9 mm. Therefore, changes in the probe-to-sample distance only impact the precision of surface potential distribution measurement and have no impact on the charge inversion calculation process.

Supplementary Note 7 | The CE mechanism of the identical PTFE

The PTFE-PTFE couple generated a triboelectric output of 1.84 V, although the residual surface charge on PTFE was eliminated before testing. Usually, triboelectricity is generated between relatively tribo-positive and tribo-negative materials. CE between two identical materials also exists and was reported previously by some scholars [8-10]. For example, *Apodaca* et al. found that this behavior appears in poly(propylene), poly(styrene), Teflon, poly(vinyl chloride), and poly(dimethylsiloxane) (PDMS), which seems to be generic to non-elemental insulators [8]. *Xu* et al. pointed out that this phenomenon originated from the "curvature effect". That is, CE of two pieces of chemically identical materials results in concave surfaces being positively charged and convex surfaces being negatively charged [9]. The surface energies of different curved surfaces would differ, resulting in a change in surface states. Electrons could transfer from one material surface to the other with shifted surface states during contact. Thus, the existence of micro/minor curved surface breaks the symmetry between the two sides, changes the surface state energy levels and causes an electron transition.

Supplementary Note 8 | Surface potential/charge tuning of PTFE

The surface potential/charge control of PTFE could be achieved by adjusting the corona discharge duration time [11]. In order to achieve a regulated introduction of ions through corona discharge for an extended duration, the separation between the metallic grid electrode and the sample was adjusted to 5 mm, and the high voltage applied to the tip electrode was reduced by roughly 500 V (8 kV, -7.5 kV). Supplementary Fig. 16 gives the relationship between the corona discharge (negative or positive polarity) duration time and the surface potential of PTFE. The negative and positive surface potential progressively rises from -271V and 286V after 3 min injection to -1706V and 1946V after 15 min injection, respectively, indicating the surface potential/charge could be regulated by adjusting the deposition duration time. The surface potential distribution in Supplementary Fig. 17 confirms that the homogeneously surface charge injection can be achieved.

It is important to note that the intensity of corona discharge is influenced by various elements, including external voltage and electrode structure. As a result, there is a specified level of uncertainty regarding the quantity of particles generated during a specific timeframe. Therefore, the actual surface potential/charge needs to be determined by measurements and inversion calculations. The three-electrode charge deposition method is a controllable and uniform surface charge control strategy, which could achieve controllable adjustment of the surface charge density on triboelectric materials combined with the proposed visualization and standardized quantification method.

Supplementary Note 9 | Surface Trap Solution Method

According to the isothermal surface potential decay (ISPD) theory, the surface trap energy level parameters can be obtained by measuring the intrinsic potential decay of the material. The sample charged by the "tip-grid-plane" electrode demonstrated even surface charge distribution. According to the "double injection" model proposed by G. Chen *et al.*[12], the charge distribution after the corona charge will appear as shown in Supplementary Fig. 64. Among them, charges with the same polarity as the corona voltage are distributed on the upper surface of the sample, and the injection depth is about 1~2 μm. On the lower surface of the sample close to the ground electrode, opposite polarity charges with the opposite polarity to the applied voltage.

Assume that the charges are uniformly distributed within δ from the surface. The positive and negative charges densities are ρ^+ and ρ^-, respectively, whose value is approximately equal, denoted as ρ. The charge density in the central area is minimal and can be ignored. Therefore, the potential on the sample surface can be expressed as,

$$\varphi_s = \frac{1}{\varepsilon_0 \varepsilon_r}\left(\int_0^{\delta} x\rho_+ dx + \int_{L-\delta}^{L} x\rho_- dx\right) \tag{12}$$

The expression for the surface potential could be obtained by solving the above integral,

$$\varphi_s = \frac{L\delta\rho}{\varepsilon_0 \varepsilon_r} \tag{13}$$

The carriers escape from the trap to form a current, which is externally manifested as the decay of the surface potential over time. The relationship between them can be expressed as,

$$\frac{\mathrm{d}\varphi_s}{\mathrm{d}t} = \frac{L\delta}{\varepsilon_0 \varepsilon_r}\frac{\mathrm{d}\rho}{\mathrm{d}t} = \frac{L\delta}{\varepsilon_0 \varepsilon_r} q_e n_t^{'} \tag{14}$$

According to the ISPD theory, the total number of electrons (or holes) emitted from the upper half (lower half) of the forbidden band to the conduction band is,

$$n_t^{'} = f_0 N(E_T)\frac{k_B T}{t} \tag{15}$$

Therefore, combining equations (14) and (15), the electron (or hole) traps density $N(E_T)$ can be obtained from the surface potential decay curve,

$$N(E_T) = \frac{\varepsilon_0 \varepsilon_r t}{q_e f_0 k_B T \delta L}\frac{\mathrm{d}\varphi_s}{\mathrm{d}t} \tag{16}$$

where ε_0 is the vacuum dielectric constant, ε_r is the relative dielectric constant of the sample, q_e is the unit charge, L is the thickness of the sample, T is the temperature, and k_B is Boltzmann's constant. f_0 is the initial occupancy rate of electron (or hole) trap. Since f_0 is difficult to measure

experimentally, in order to simplify the calculation, it is assumed that $f_0 \approx 1$, that is, the initial occupancy rate of the trap is 100%, and the density of the trap is the density of trapped charges.

Traps are artificially separated into two types: deep traps and shallow traps, considering the various energy levels of traps where de-trapping carriers are positioned. The carrier de-trapping in the two types of traps corresponds to the different processes of material surface potential decay. Therefore, the measured potential decay curve can be fitted with a double exponential function:

$$\varphi_s(t) = a_1 \exp(-b_1 t) + a_2 \exp(-b_2 t) \tag{17}$$

where a_1, b_1, a_2, b_2 are the fitting parameters.

The electron and hole trap energy level distribution can be obtained by combining the fitted curve with equation (17). The negative polarity potential decay curve corresponds to the electron trap, and the positive polarity potential decay curve corresponds to the hole trap.

Supplementary Figures

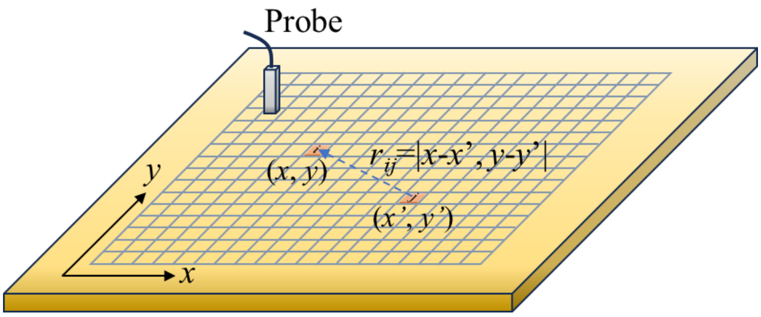

Supplementary Fig. 1 | Schematic diagram of potential contribution of point *j* to point *i*.

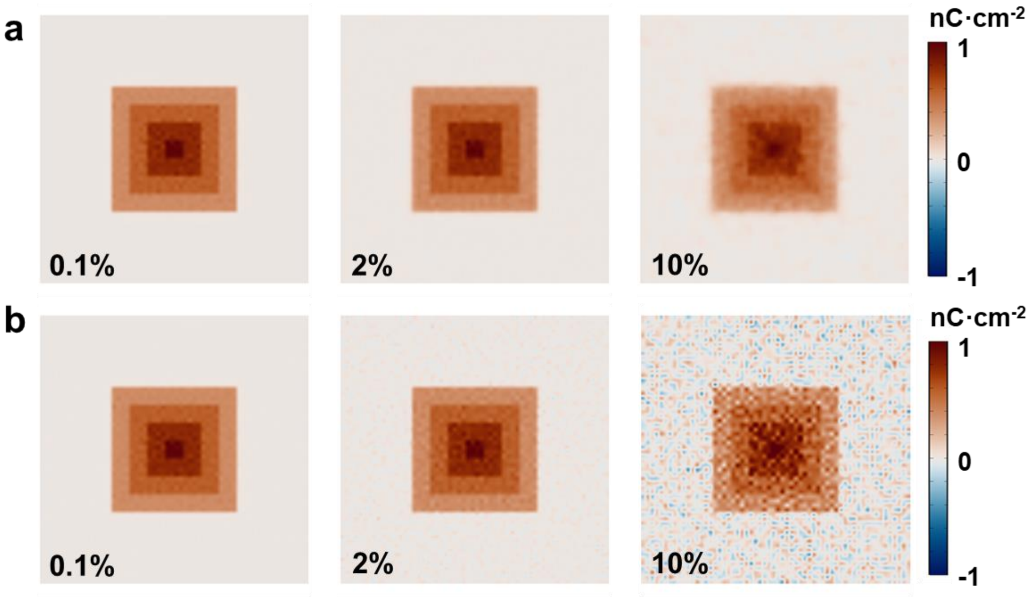

Supplementary Fig. 2 | The surface charge distributions of *Case 1* under different Gaussian noise level conditions. **a**, Visualization and standardized quantification (VSQ) method. **b**, Charge simulation (CS) method. (Area range: 30mm×30mm)

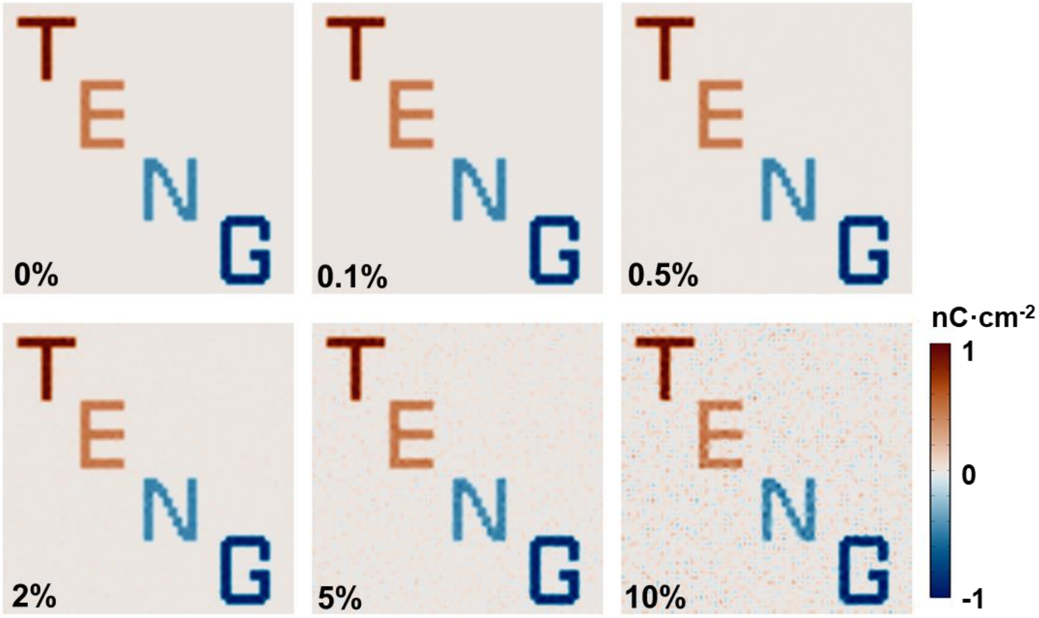

Supplementary Fig. 3 | The surface charge distributions of *Case 2* under different Gaussian noise level conditions obtained by the charge simulation (CS) method. (Area range: 30mm× 30mm)

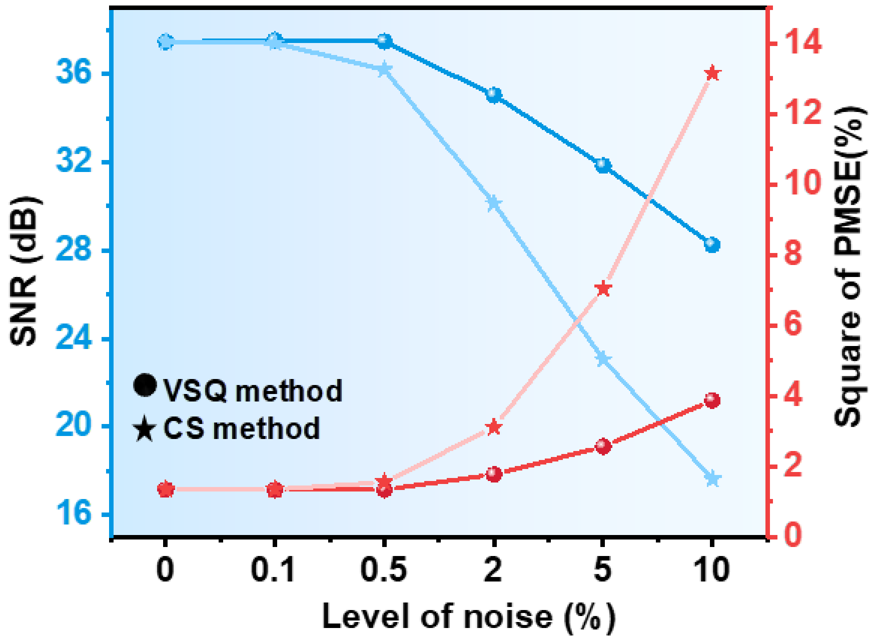

Supplementary Fig. 4 | The signal to noise ratio (SNR) and peak mean square error (PMSE) of the visualization and standardized quantification (VSQ) and charge simulation (CS) method under different Gaussian noise level conditions (*Case 1*).

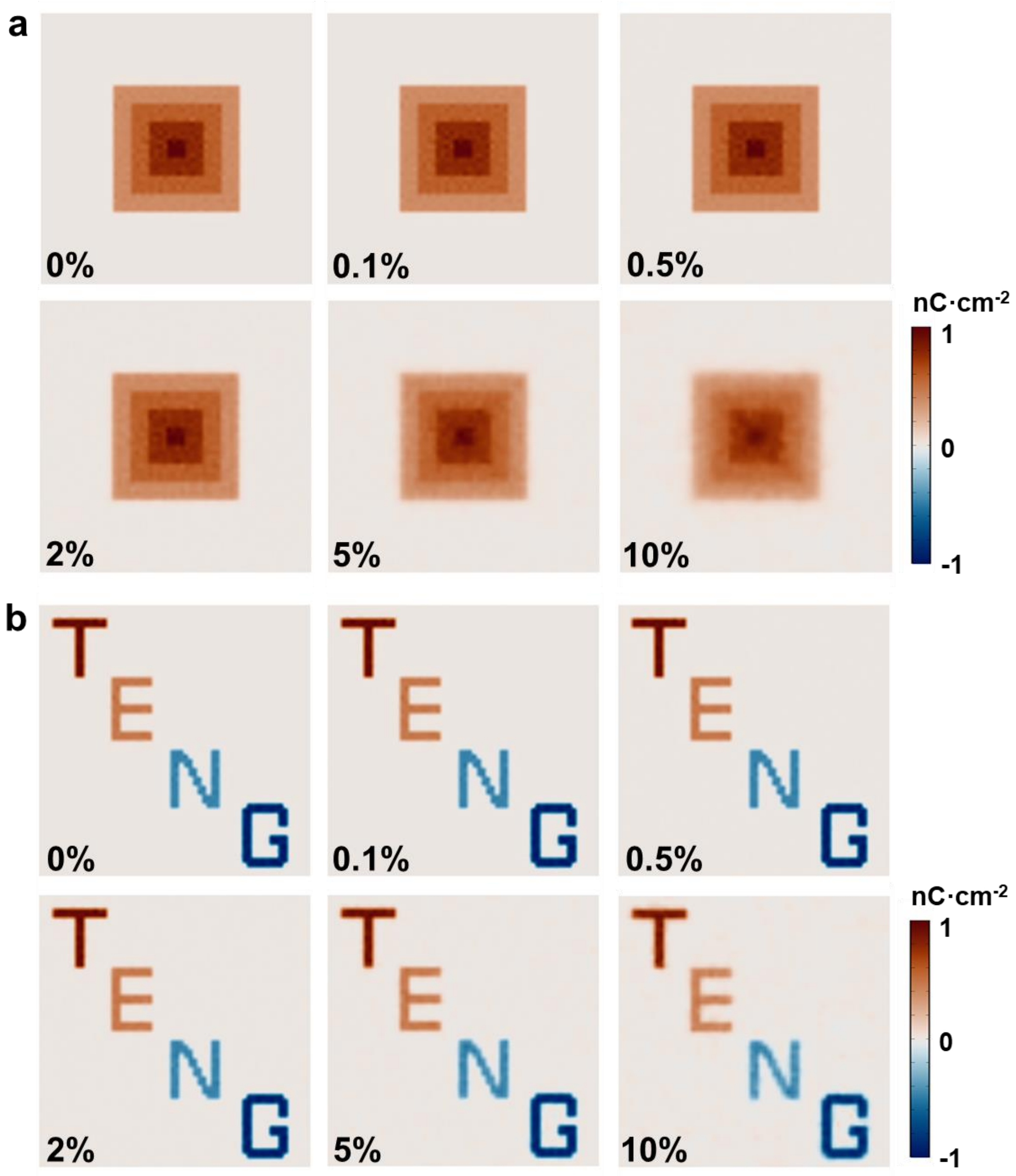

Supplementary Fig. 5 | The surface charge distributions obtained by the generalized cross validation (GCV) approach based visualization and standardized quantification (VSQ) method. a, *Case 1*. **b**, *Case 2*. (Area range: 30mm×30mm)

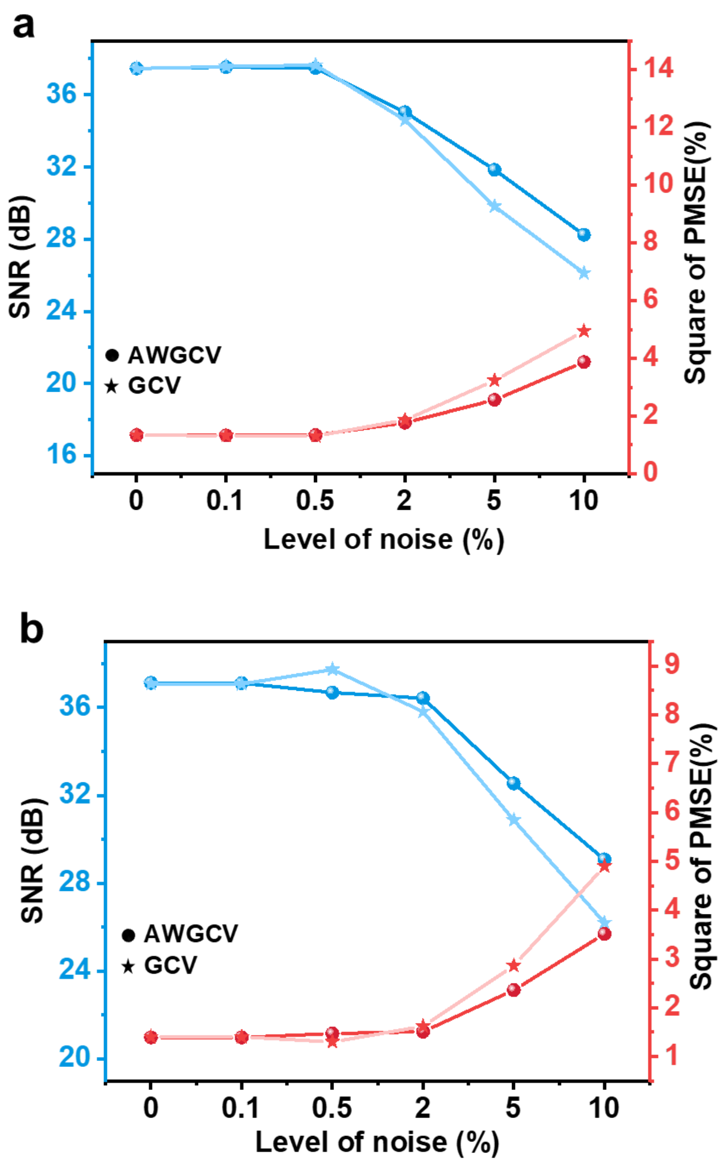

Supplementary Fig. 6 | The signal to noise ratio (SNR) and peak mean square error (PMSE) of the adaptive weight generalized cross validation (AWGCV) and generalized cross validation (GCV) approach based VSQ method under different noise levels. a, *Case 1*. **b**, *Case 2*.

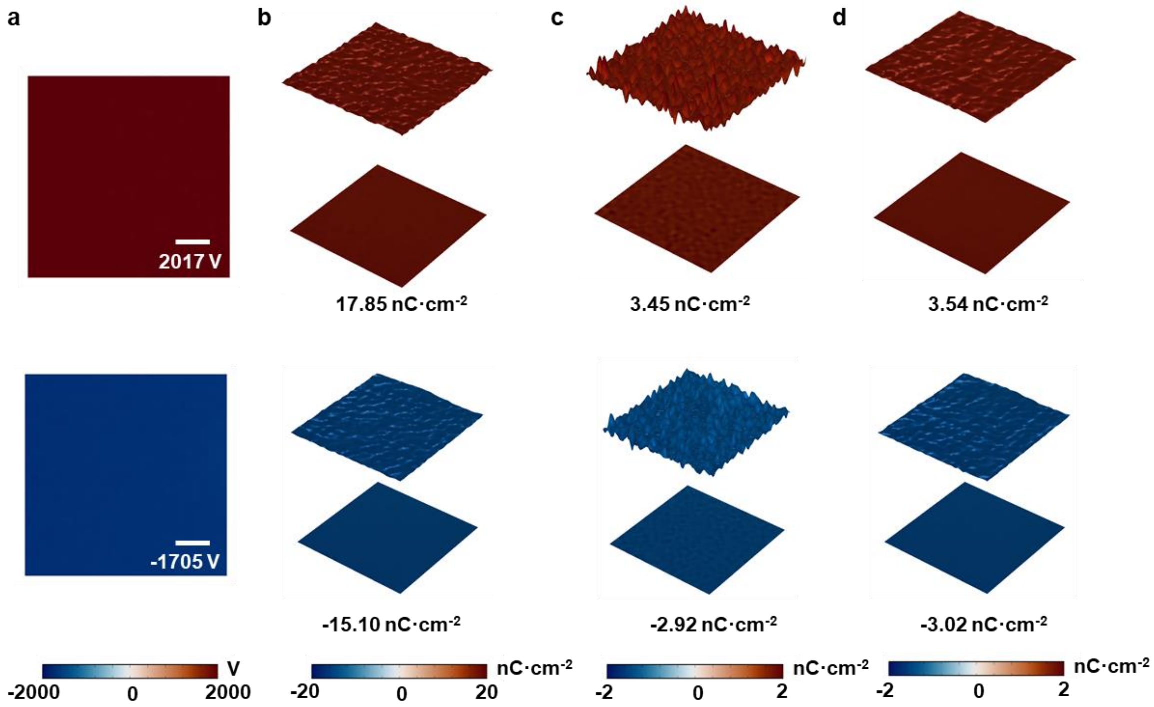

Supplementary Fig. 7 | Comparison of the traditional and proposed surface charge visualization and standardized quantification (VSQ) method. a, Surface potential distribution of the uniformly charged sample. **b**, Surface charge distribution and density values obtained by traditional capacitor model. **c**, Surface charge distribution and density values obtained by the charge simulation (CS) method. **d**, Surface charge distribution and density values obtained by the proposed VSQ method. (Sacle bar: 3 mm).

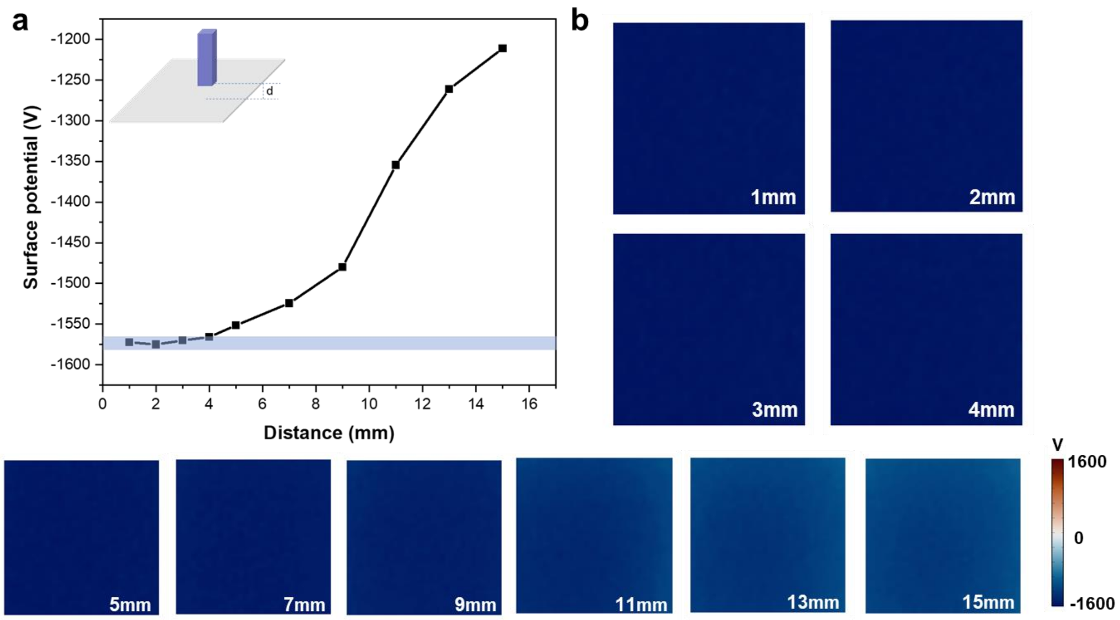

Supplementary Fig. 8 | The influence of probe-to-surface distance (*d*) on the surface potential measurement accuracy. a, The relationship between detected surface potential and *d*. **b**, The surface potential distribution of the 15×15 mm PTFE sample under different probe-to-surface distances.

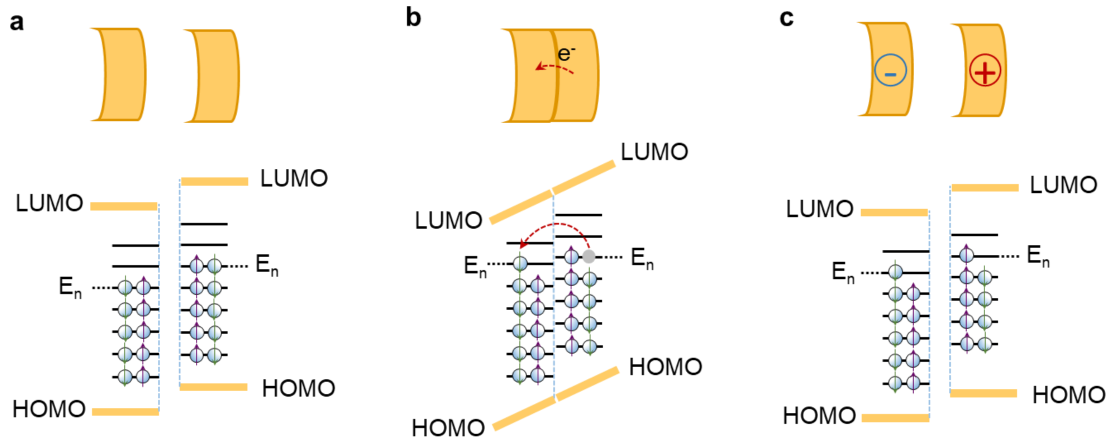

Supplementary Fig. 9 | Mechanism of the CE between identical materials of different surface curvatures. a, Charge transfer before contact. **b**, In contact. **c**, After contact.

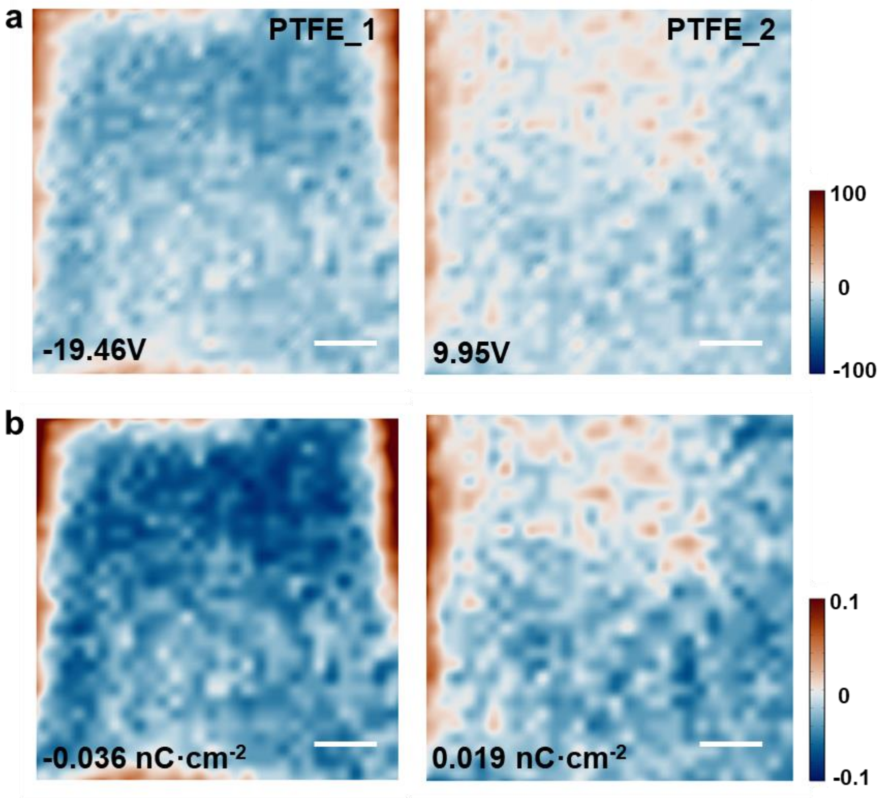

Supplementary Fig. 10 | The surface potential and surface charge of the PTFE-PTFE couple after contact electrification (CE). a, Surface potential. **b**, Surface charge. (Scale bar: 3 mm) The PTFE_1 generated the negative CE potential/charge (blue region), whereas the positive potential/charge existed in the corresponding position on PTFE_2 (the yellow region). The experimental results are consistent with the above-mentioned analysis.

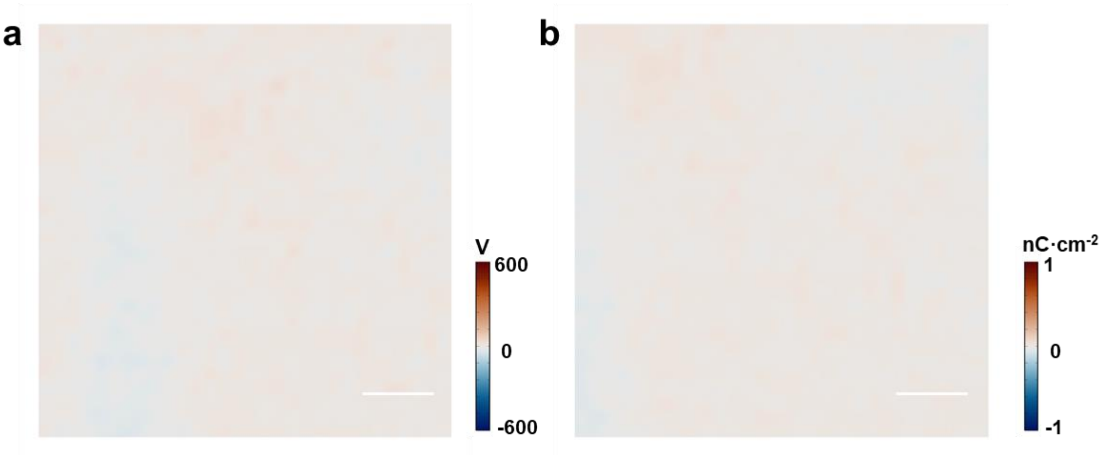

Supplementary Fig. 11 | The surface potential and surface charge distribution of the PTFE film treated by air-ionization gun. a, Surface potential. **b**, Surface charge. (Scale bar, 3 mm) The results indicate that the residual charge was successfully removed.

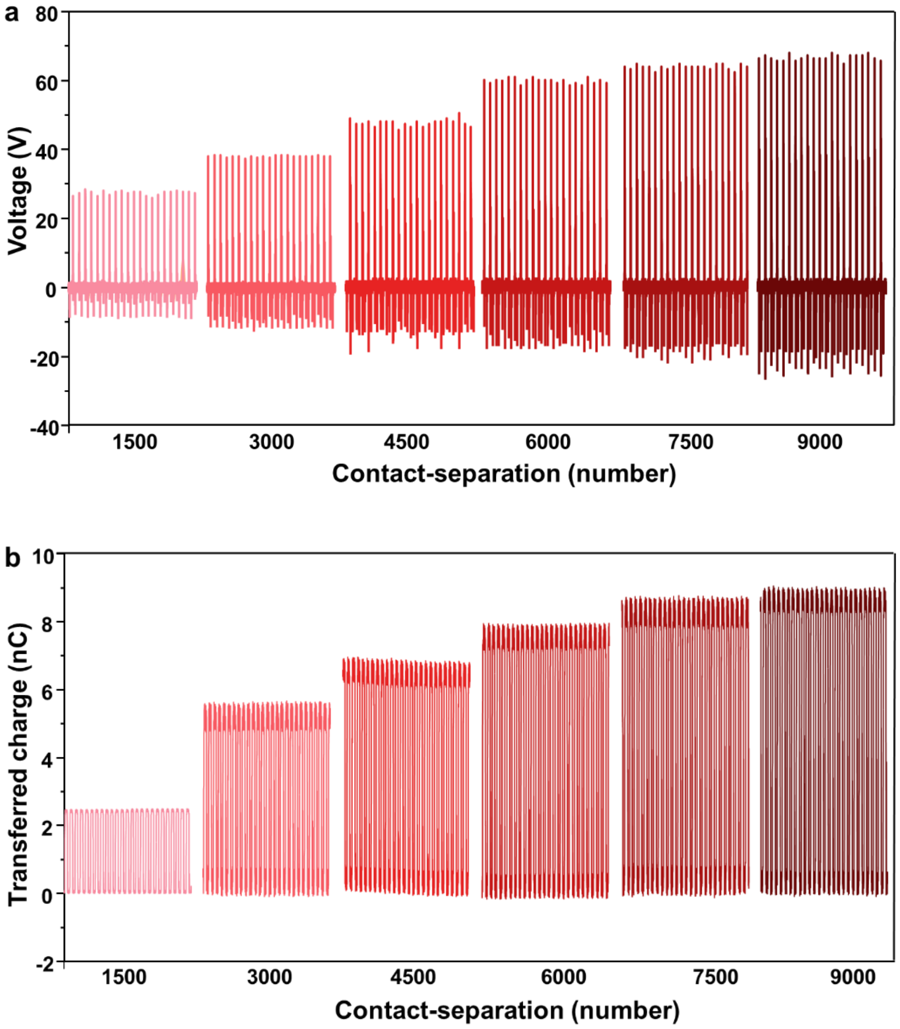

Supplementary Fig. 12 | The output properties of the PTFE-Aliuminum triboelectric pair-based TENG under different contact-separation numbers. a, Output voltage. **b**, Transferred charge. (Sample size: 2×2 cm, F=50 N)

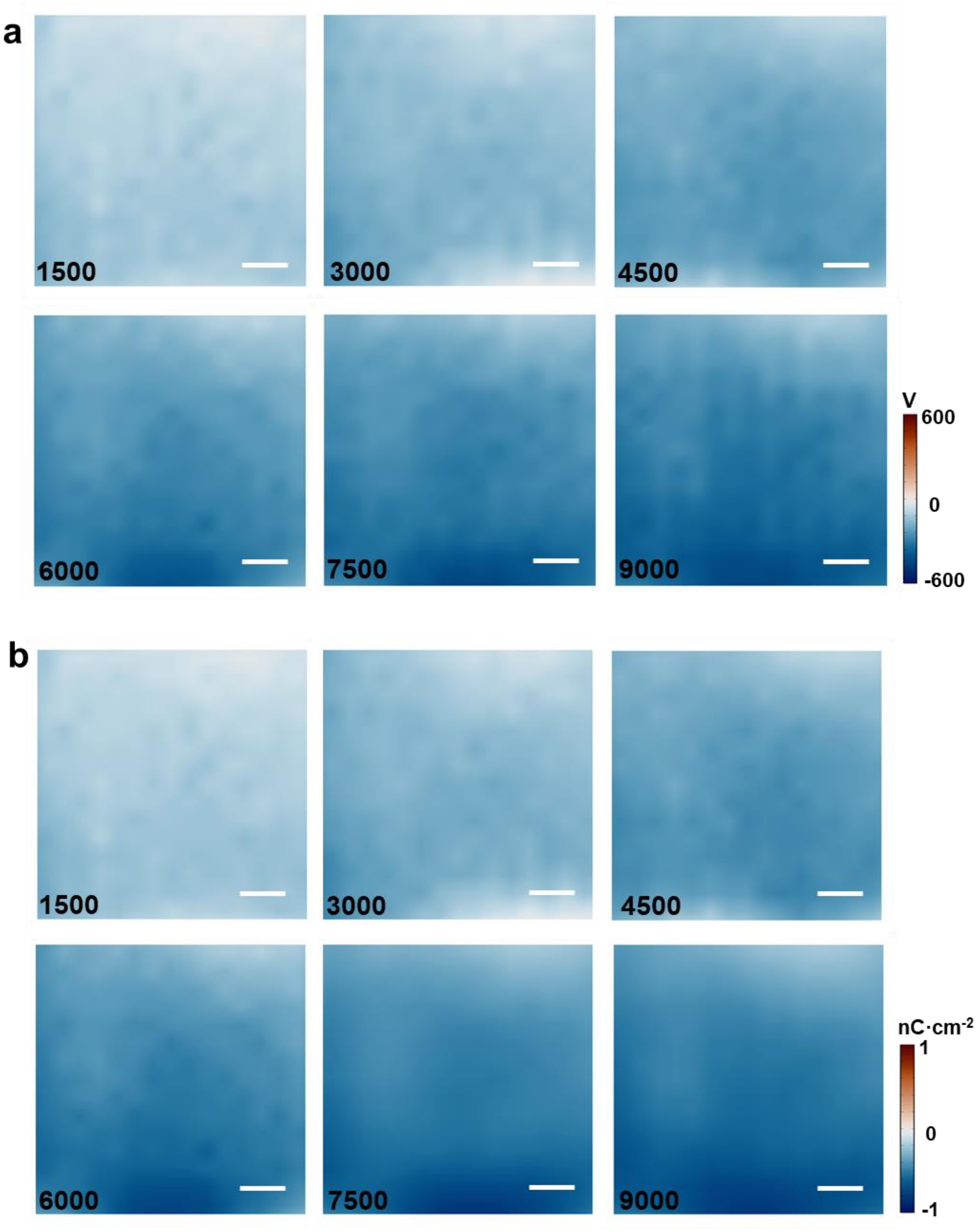

Supplementary Fig. 13 | The surface potential and surface charge distribution of PTFE-Aluminum triboelectric pair-based TENG under different contact-separation numbers. **a**, Surface potential. **b**, Surface charge. (Scale bar: 3 mm)

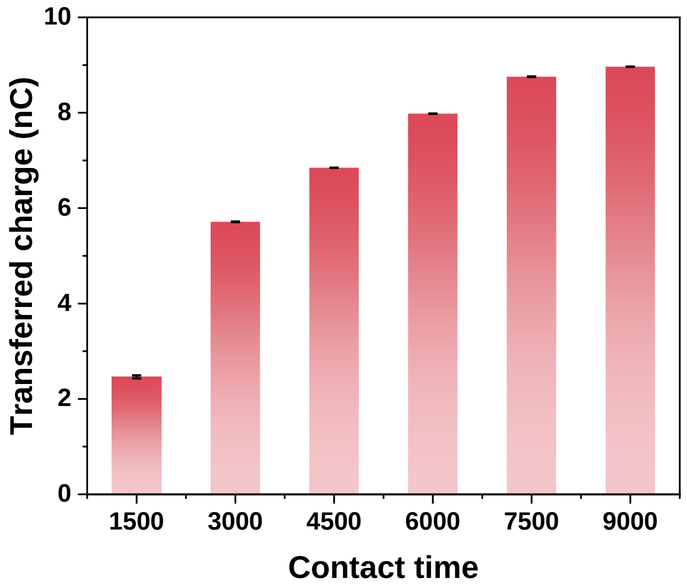

Supplementary Fig. 14 | The transferred charge of PTFE-Aluminum triboelectric pair-based TENG under different contact-separation numbers.

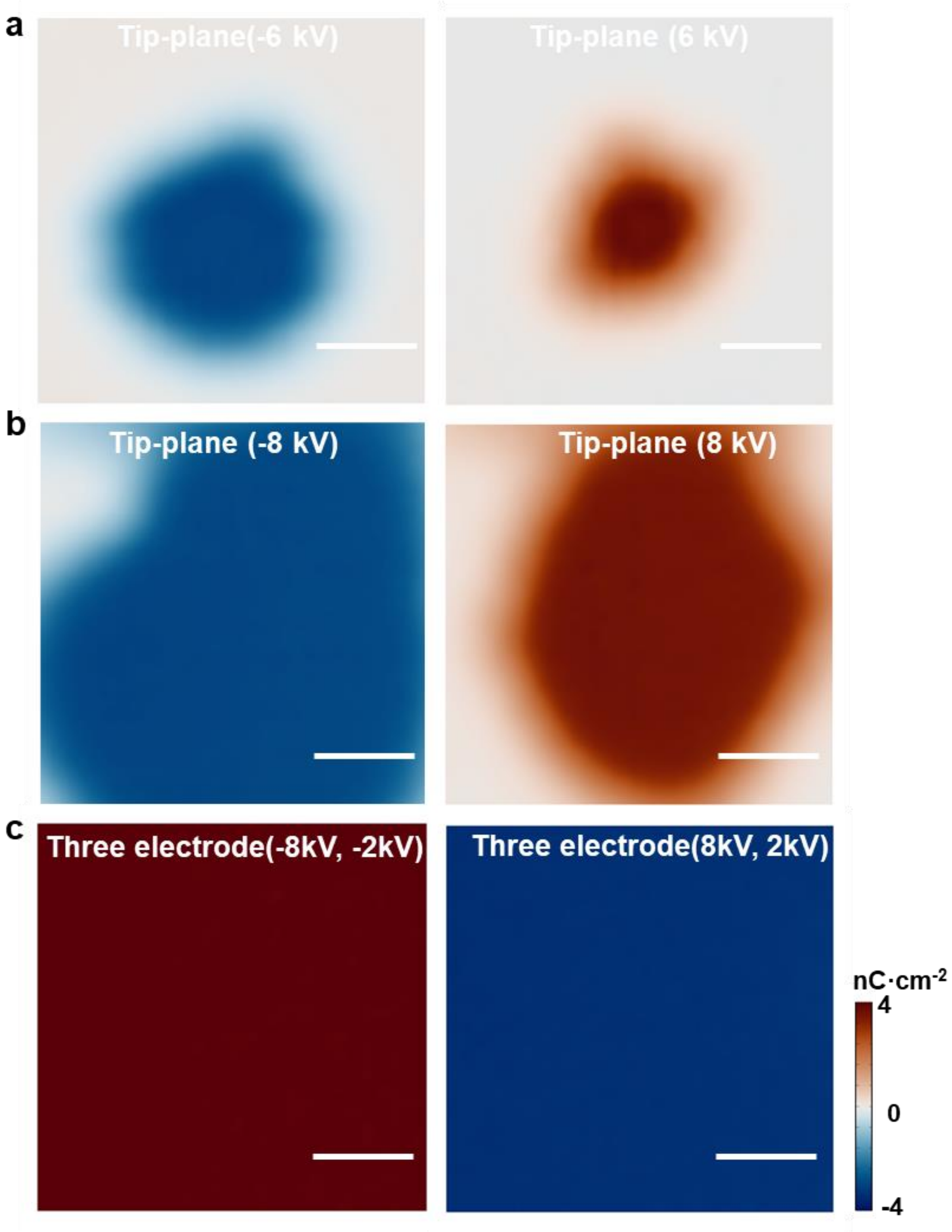

Supplementary Fig. 15 | The surface potential and charge distribution of tip-plane electrode-induced corona discharge. a, The -6 kV and 6 kV electrode system. **b**, The -8 kV and 8 kV electrode system. **c**, The three-electrode system. (Scale bar: 5 mm) The results verified that the grid electrode ensured uniform distribution of surface charge.

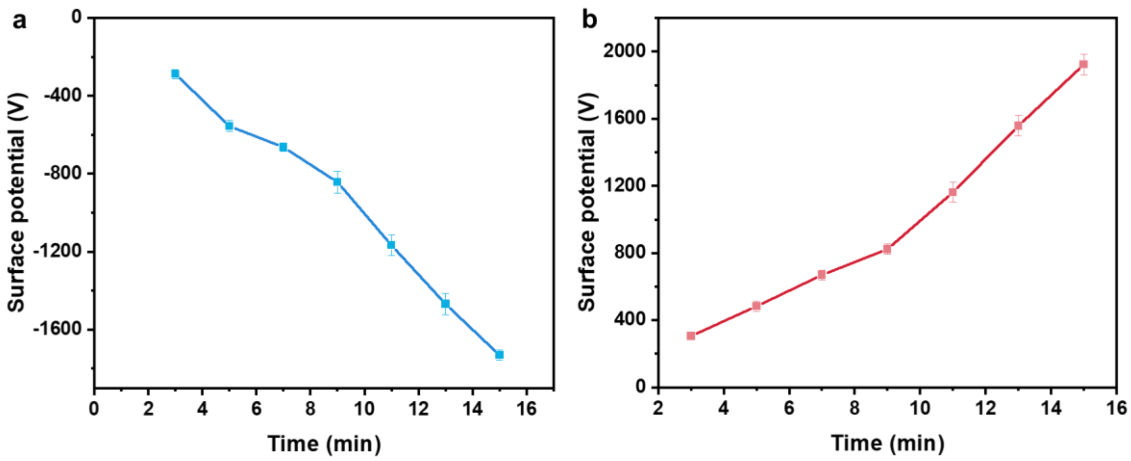

Supplementary Fig. 16 | The relationship between the corona discharge duration time and the surface potential. a, Negative polarity (-7.5 kV, -2 kV). **b**, Positive polarity (8 kV, 2 kV).

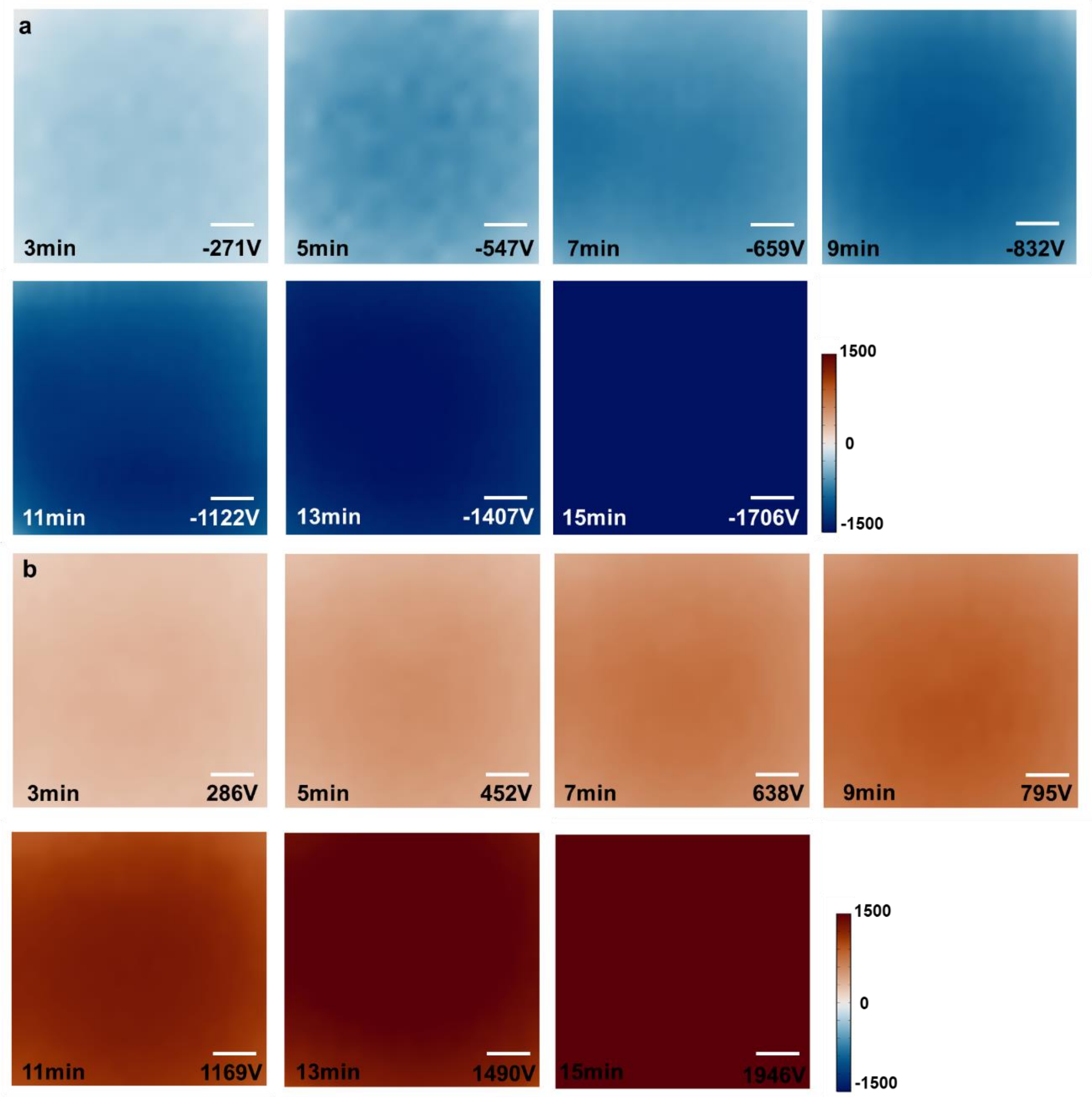

Supplementary Fig. 17 | The surface potential distribution of PTFE after different deposition durations. a, Negative polarity corona discharge. **b**, Positive polarity corona discharge. (Scale bar: 3mm).

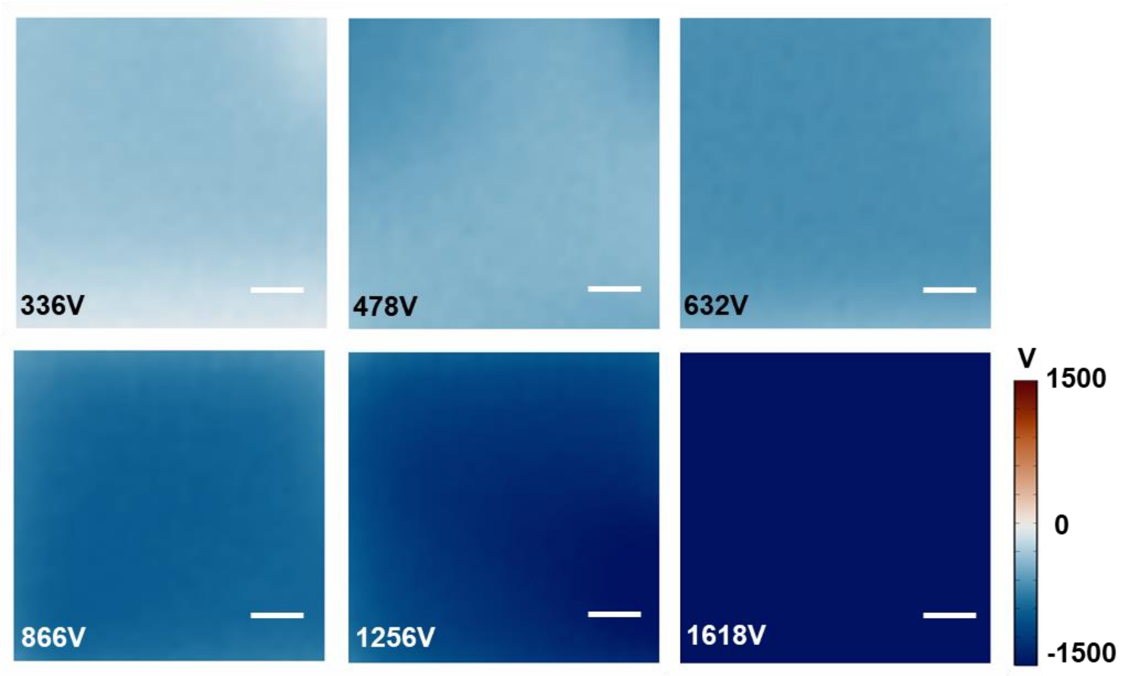

Supplementary Fig. 18 | Surface potential tuning of PTFE by depositing negative charges. (Scale bar: 3 mm)

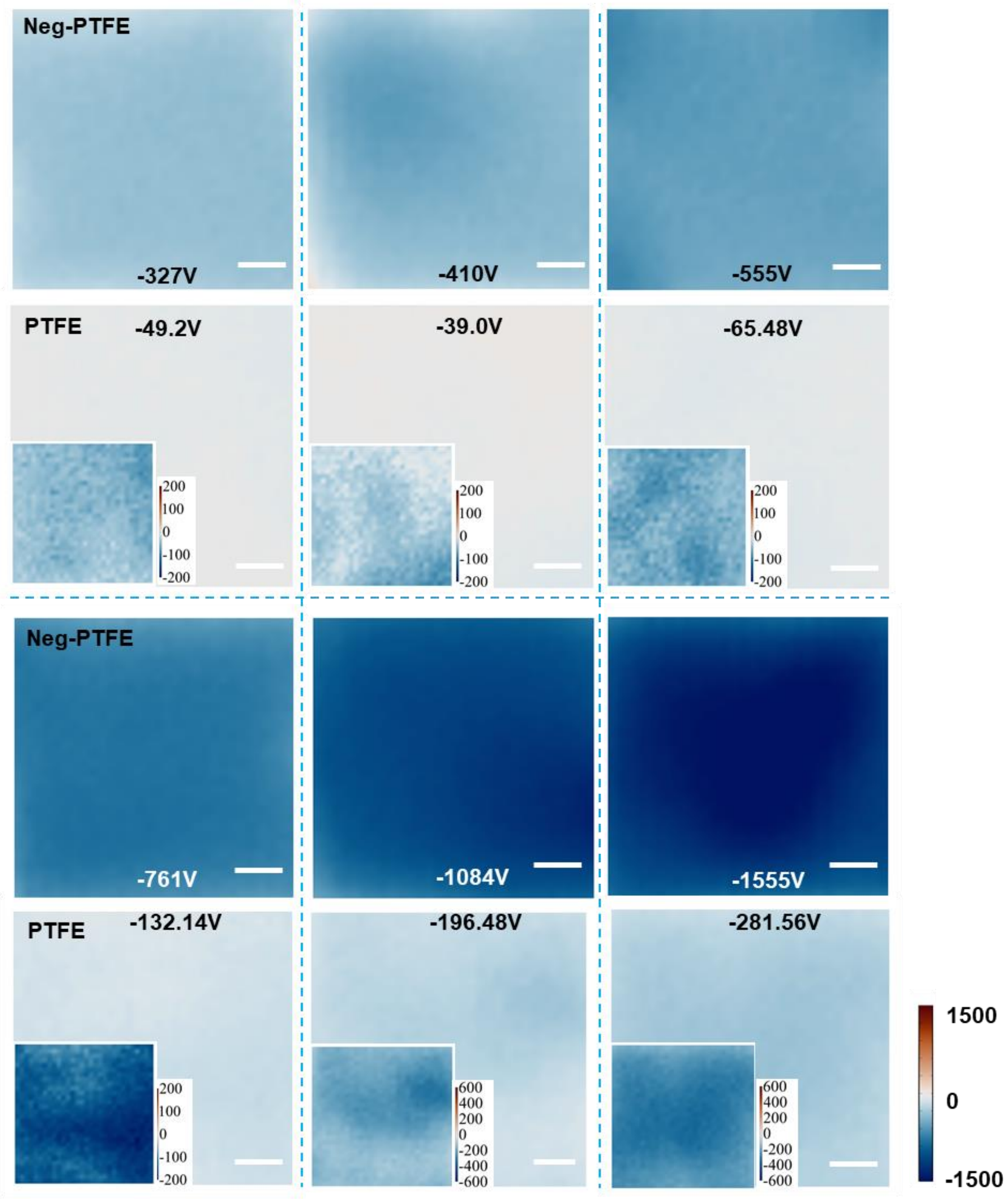

Supplementary Fig. 19 | Surface potential distribution of neg-PTFE/PTFE triboelectric pair after 5000 contact-separation cycles. (Scale bar: 3 mm)

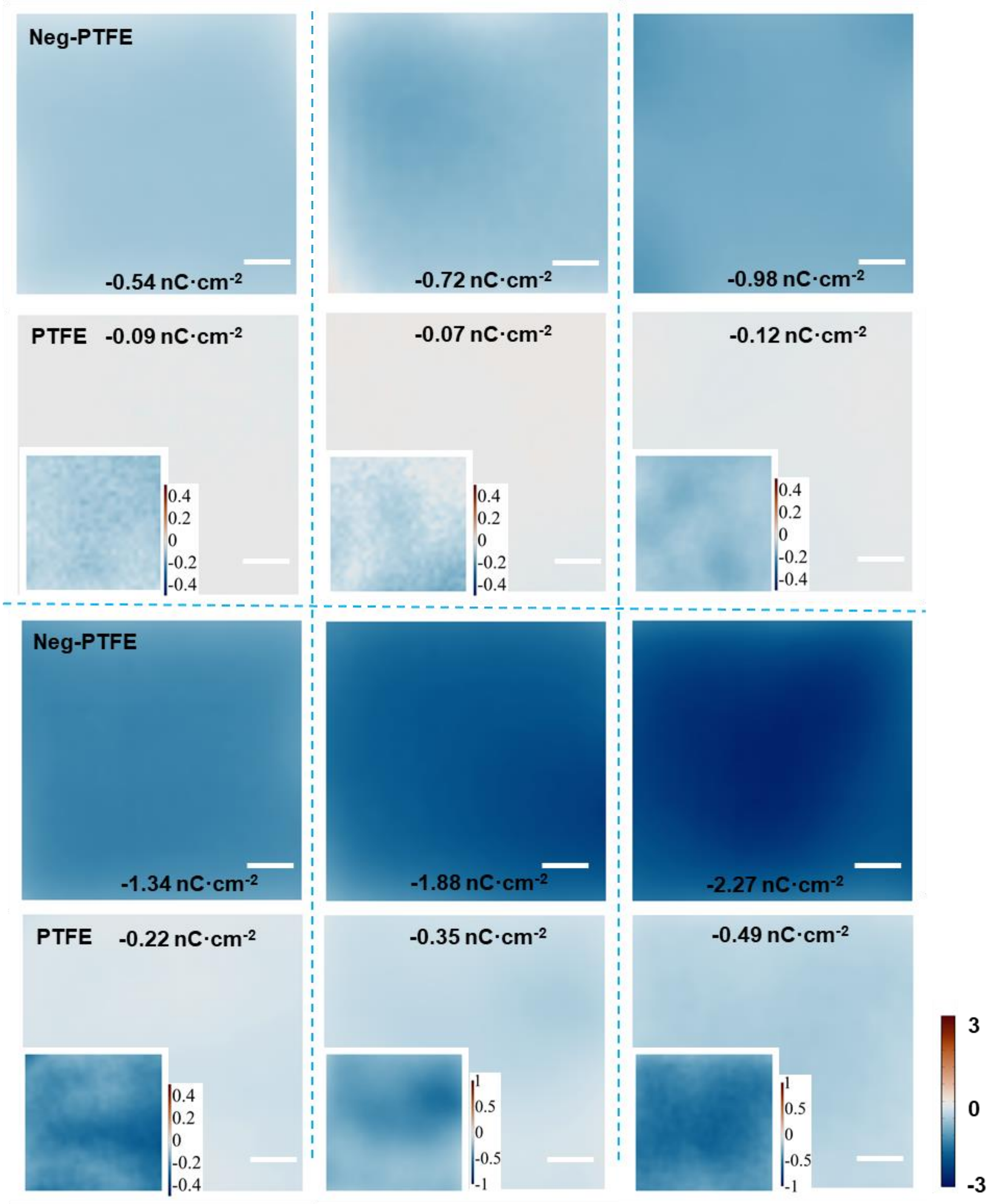

Supplementary Fig. 20 | Surface charge distribution of neg-PTFE/PTFE triboelectric pair after 5000 contact-separation cycles. (Scale bar: 3 mm)

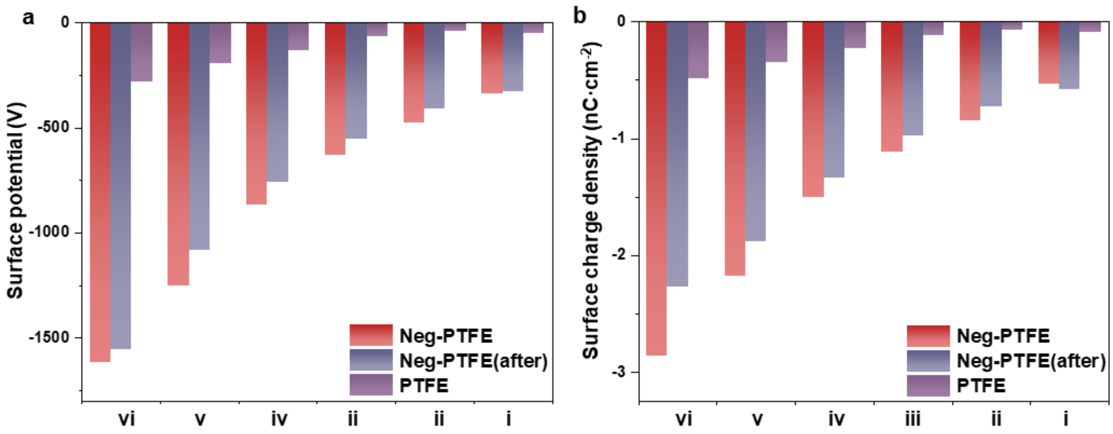

Supplementary Fig. 21 | Comparison of the surface potential and surface charge density for neg-PTFE, neg-PTFE (after operation) and PTFE (after operation). a, Surface potential. **b**, surface charge. The neg-PTFE/PTFE triboelectric pair based TENG was subjected to 5000 operational cycles.

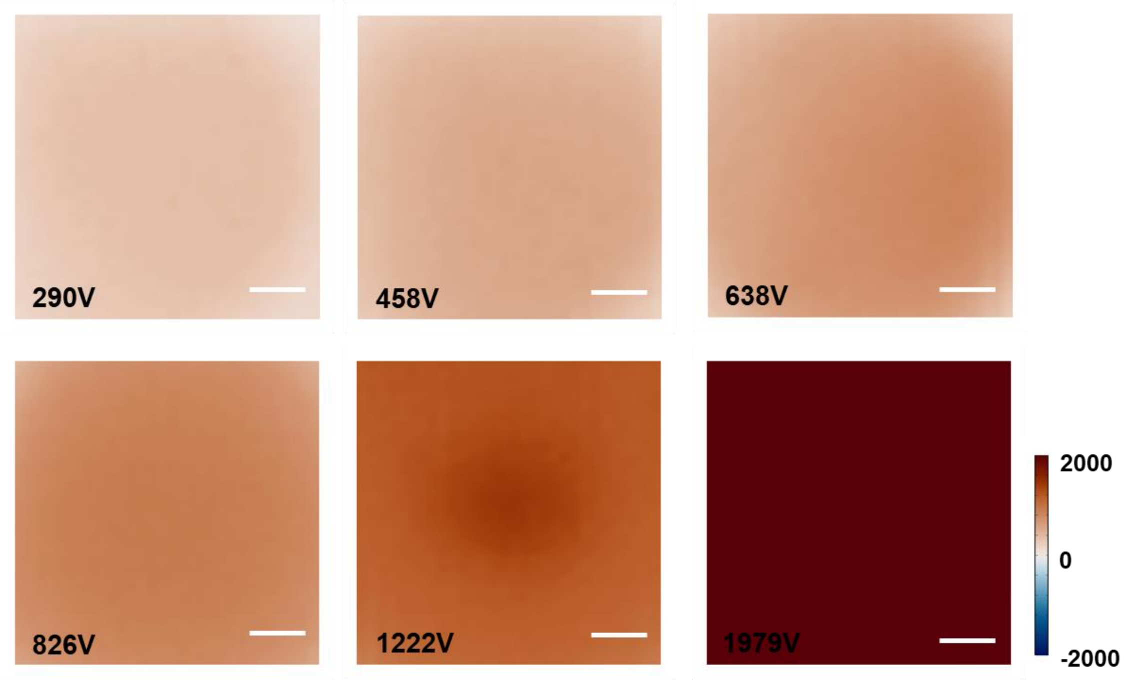

Supplementary Fig. 22 | Surface potential tuning of PTFE by depositing positive charges. (Scale bar: 3 mm)

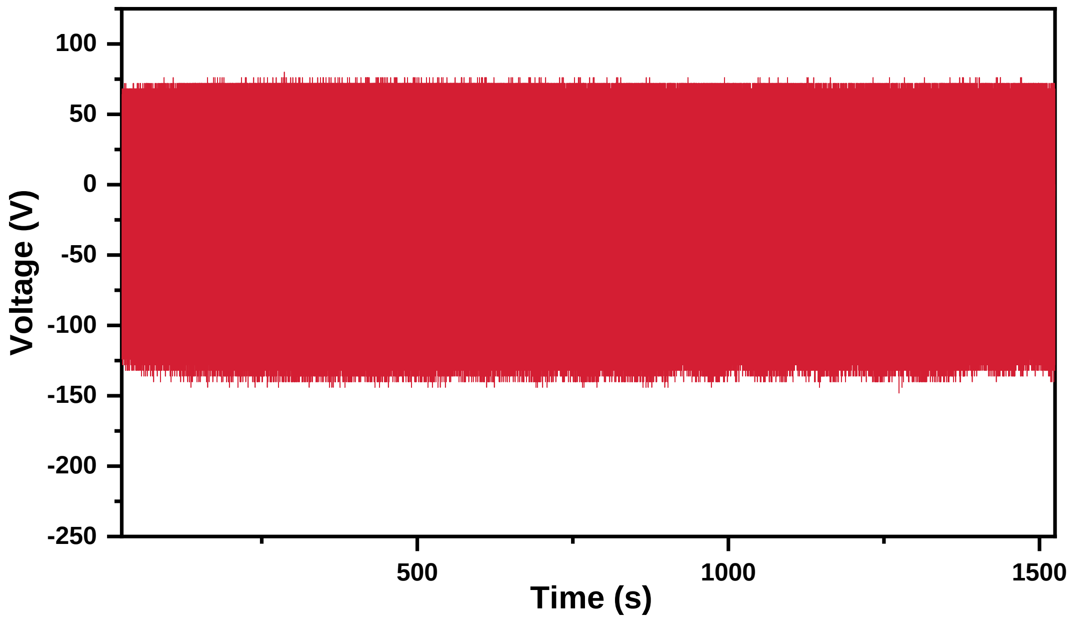

Supplementary Fig. 23 | The long-term output voltage waveform of posi-PTFE/PTFE triboelectric pair based TENG.

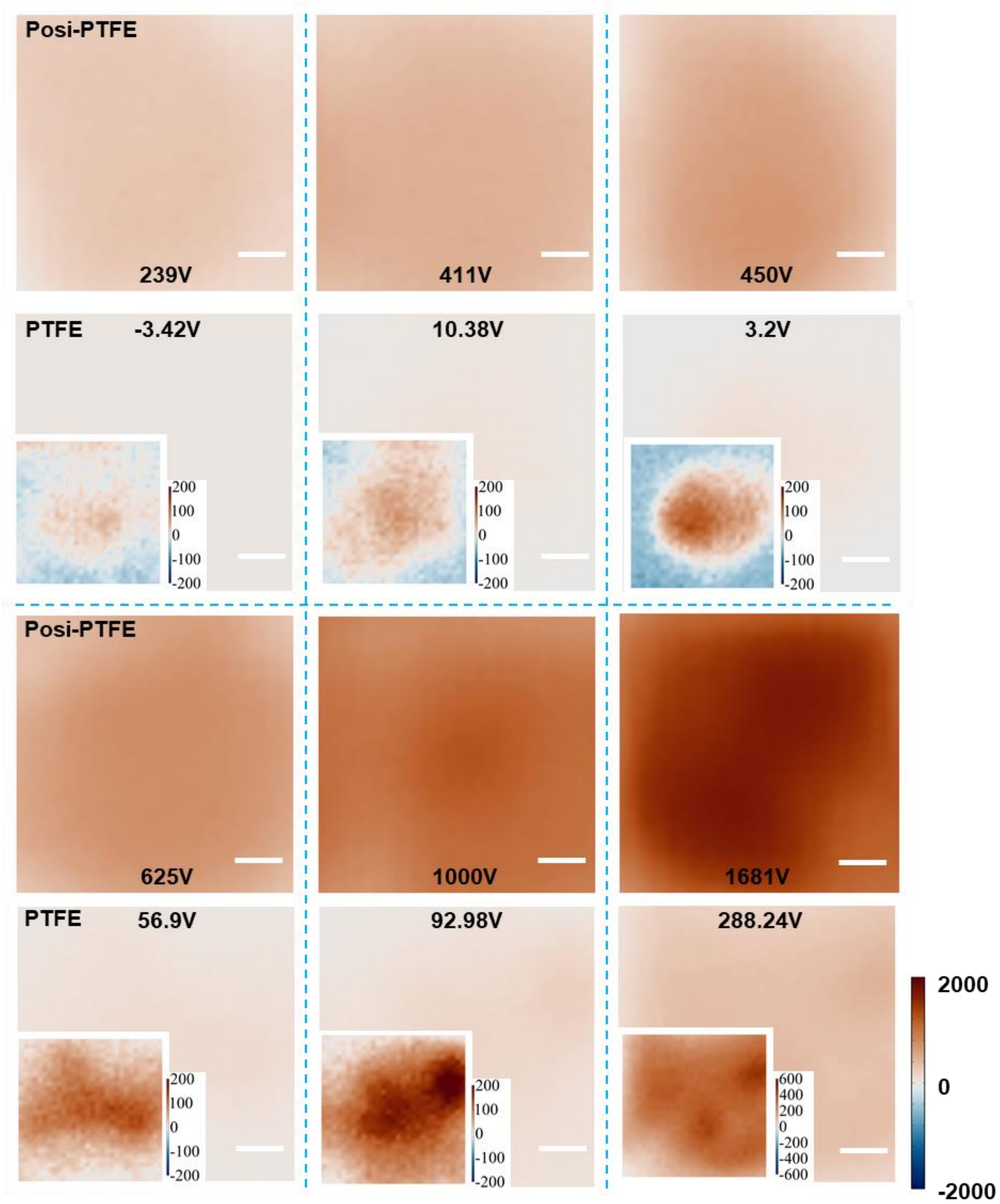

Supplementary Fig. 24 | Surface potential distribution of posi-PTFE/PTFE triboelectric pair after 5000 contact-separation cycles. (Scale bar: 3 mm)

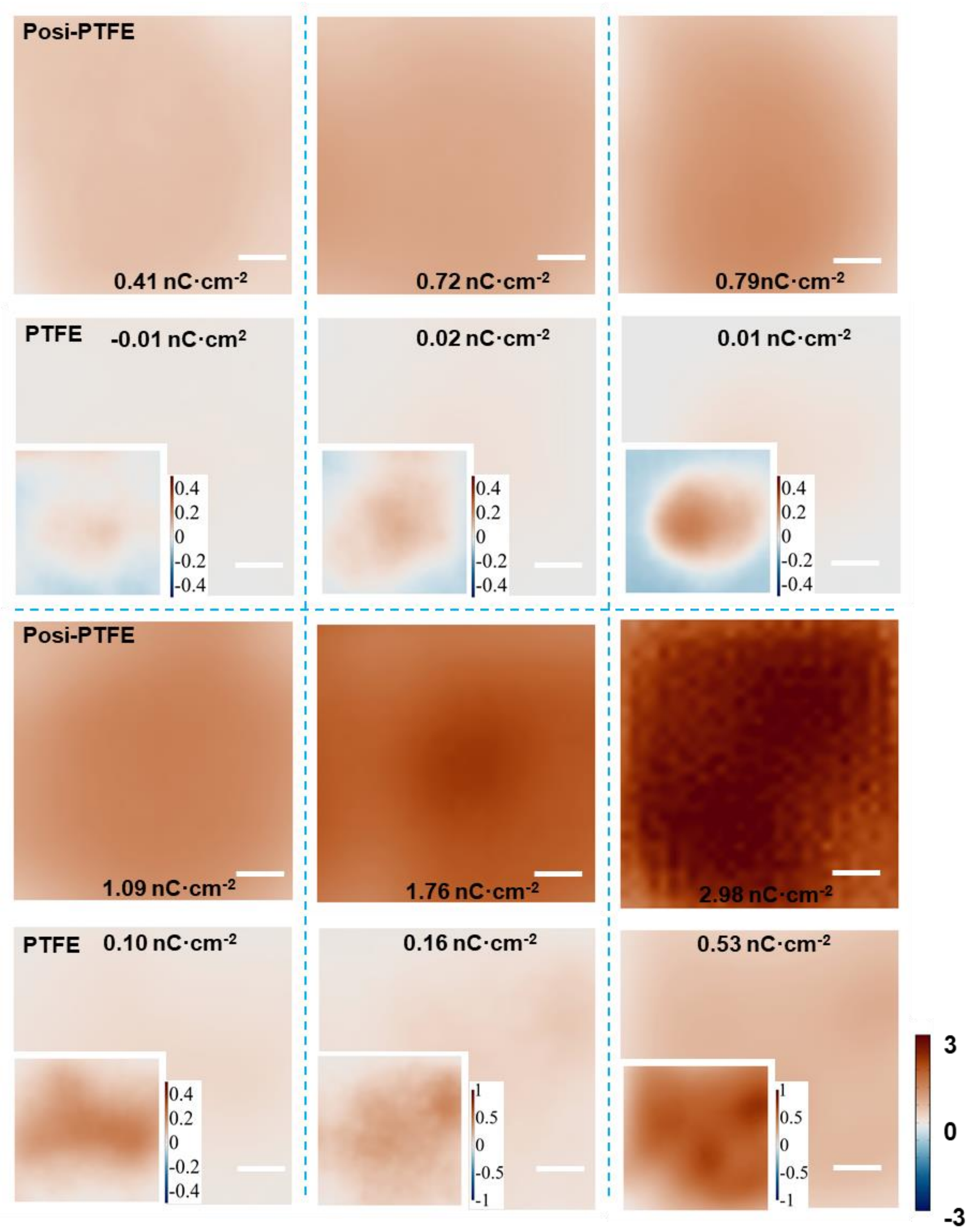

Supplementary Fig. 25 | Surface charge distribution of posi-PTFE/PTFE triboelectric pair after 5000 contact-separation cycles. (Scale bar: 3 mm)

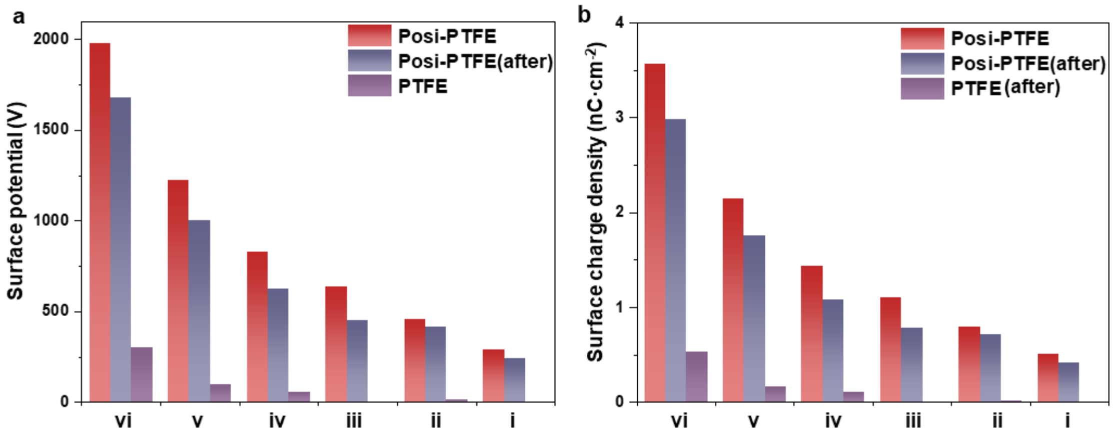

Supplementary Fig. 26 | Comparison of the surface potential and surface charge density for posi-PTFE, posi-PTFE(after operation) and PTFE(after operation). a, Surface potential. **b**, Surface charge. The posi-PTFE/PTFE triboelectric pair based TENG was subjected to 5000 operational cycles.

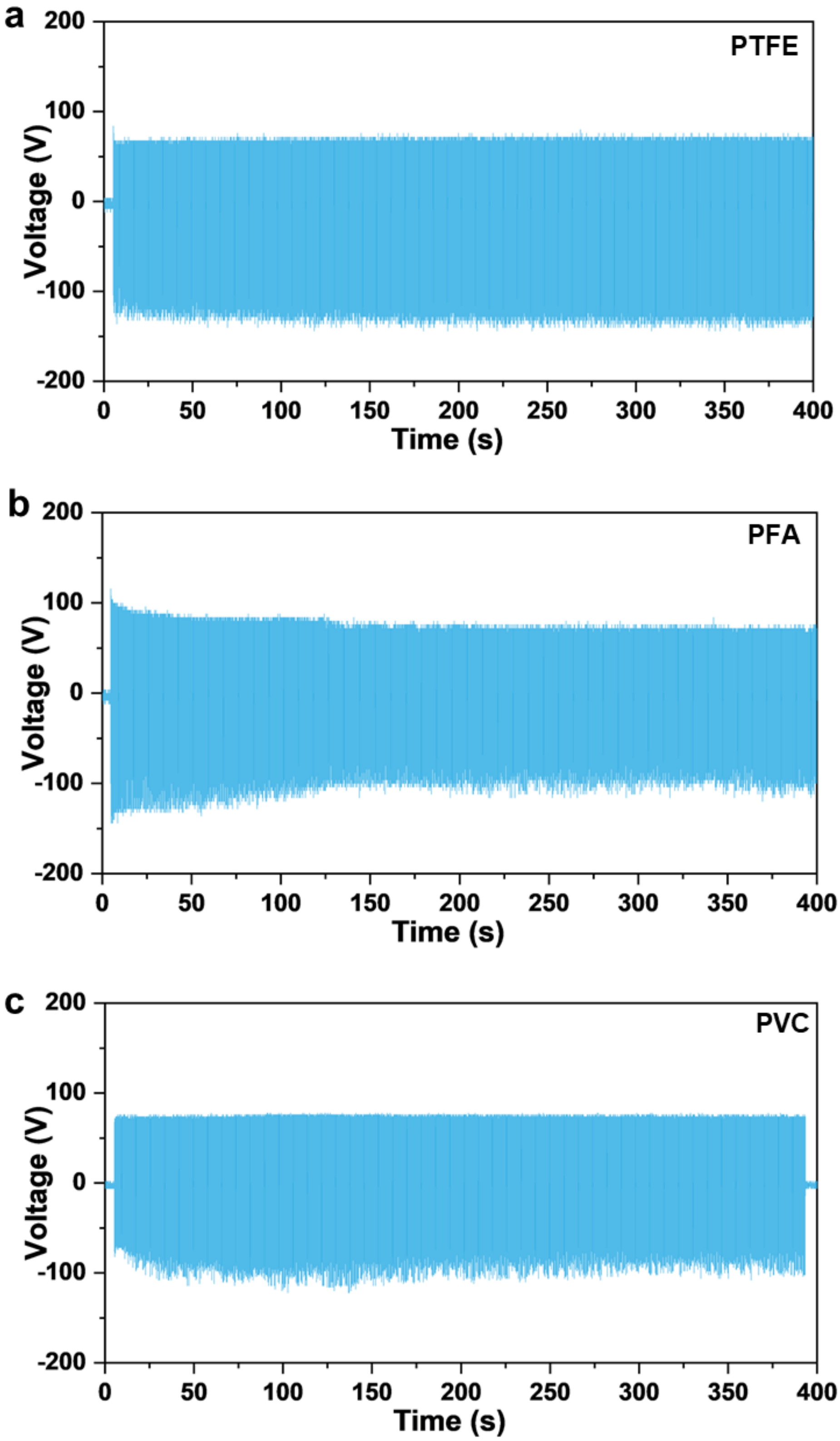
a
PTFE
Voltage (V)
200
100
0
-100
-200
Time (s)
0
50
100
150
200
250
300
350
400
b
PFA
c
PVC

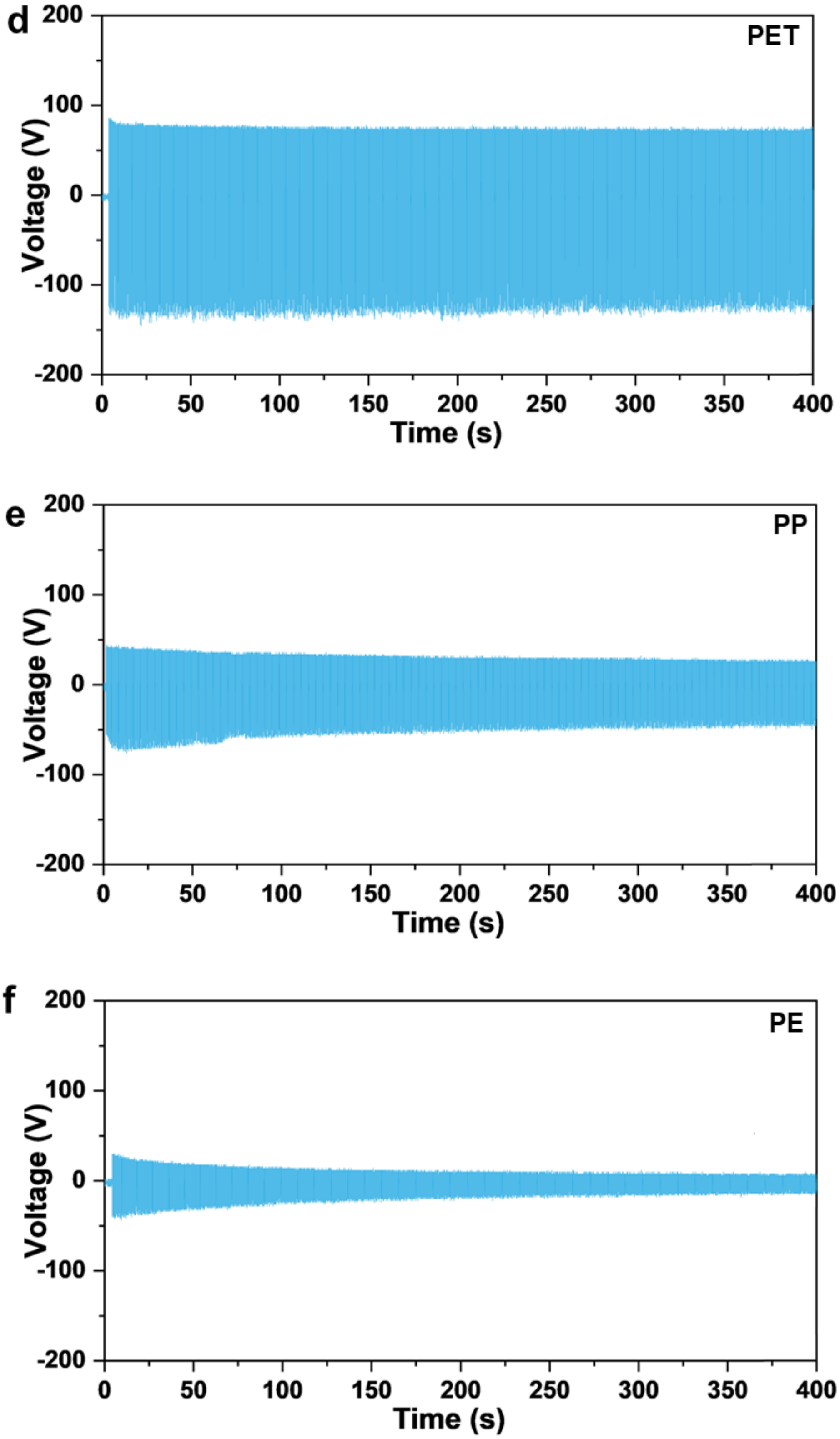
d
PET
Voltage (V)
Time (s)
e
PP
Voltage (V)
Time (s)
f
PE
Voltage (V)
Time (s)

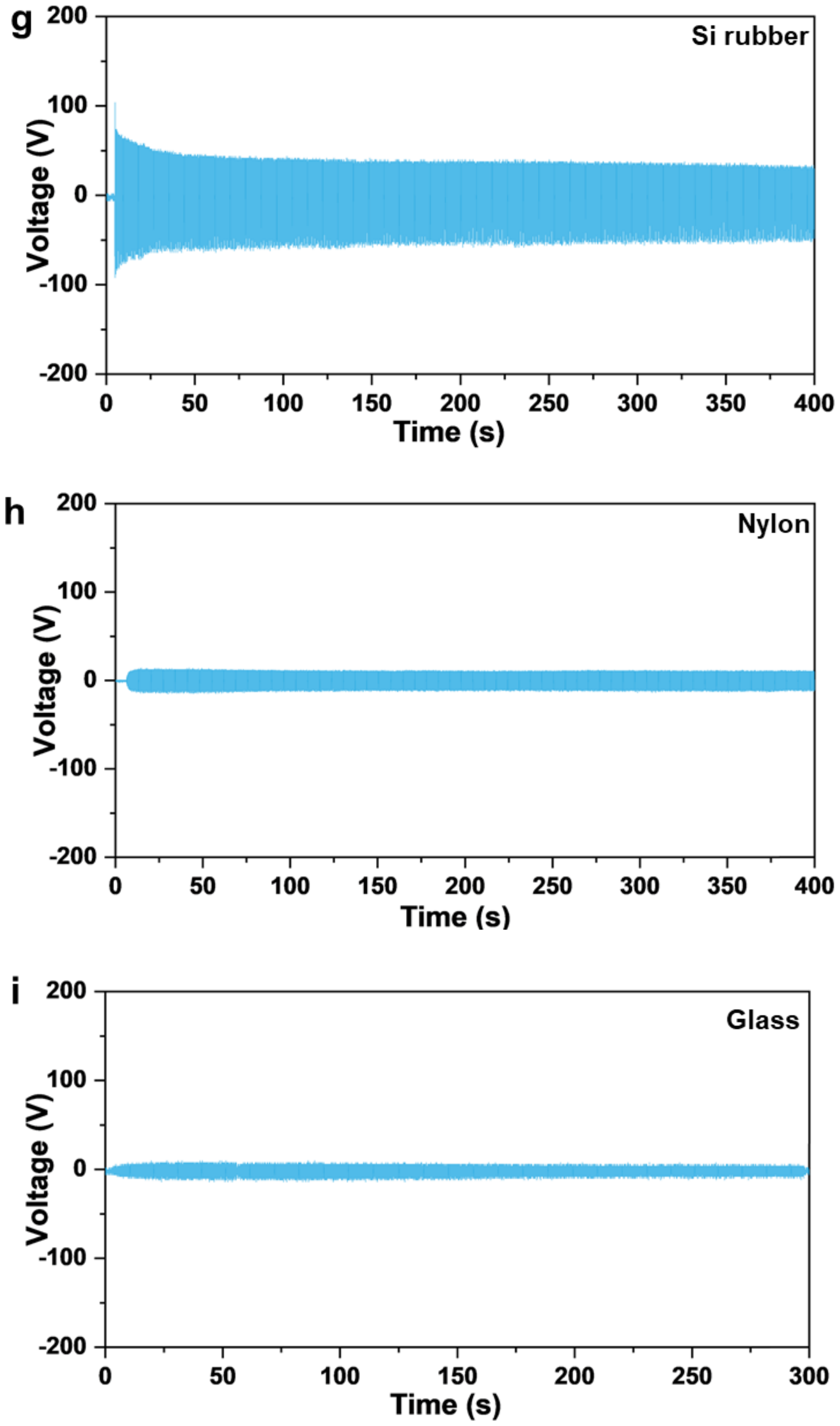

Supplementary Fig. 27 | The output voltage waveform of positive charged dielectric film based TENG. a, The posi-PTFE/PTFE triboelectric pair. **b**, The posi-PFA/PFA triboelectric pair. **c**, The posi-PVC/PVC triboelectric pair. **d**, The posi-PET/PET triboelectric pair. **e**, The

posi-PP/PP triboelectric pair. **f**, The posi-PE/PE triboelectric pair. **g**, The posi-Si rubber/Si rubber triboelectric pair. **h**, The posi-Nylon/Nylon triboelectric pair. **i**, The posi-glass/glass triboelectric pair.

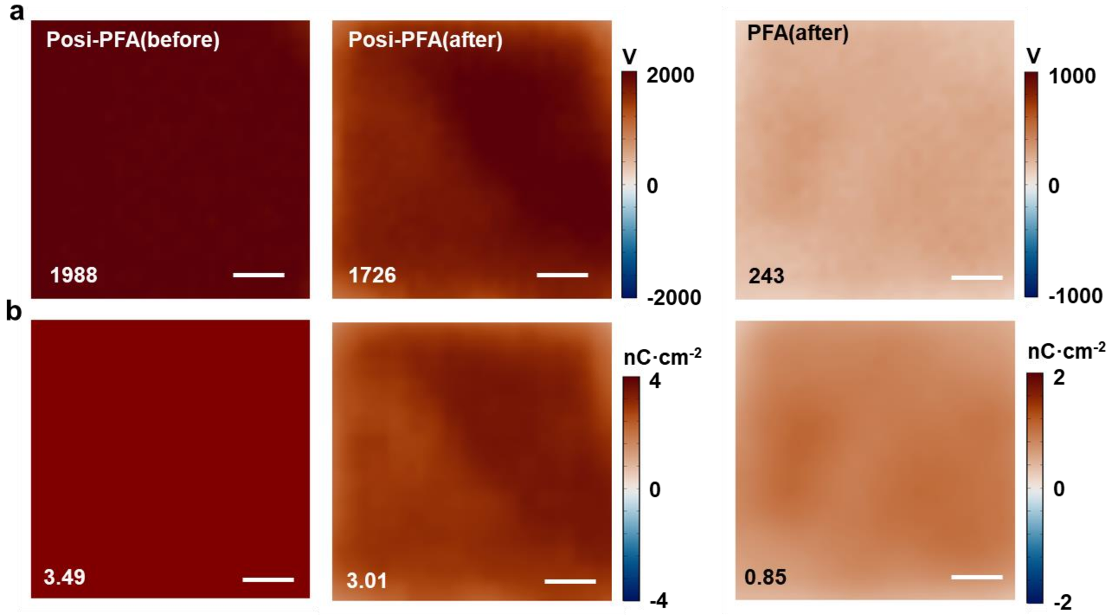

Supplementary Fig. 28 | The surface potential and surface charge distribution of the positive charged PFA based TENG before and after 5000 contact-separation cycles. a, Surface potential. **b**, Surface charge. (Scale bar: 3 mm)

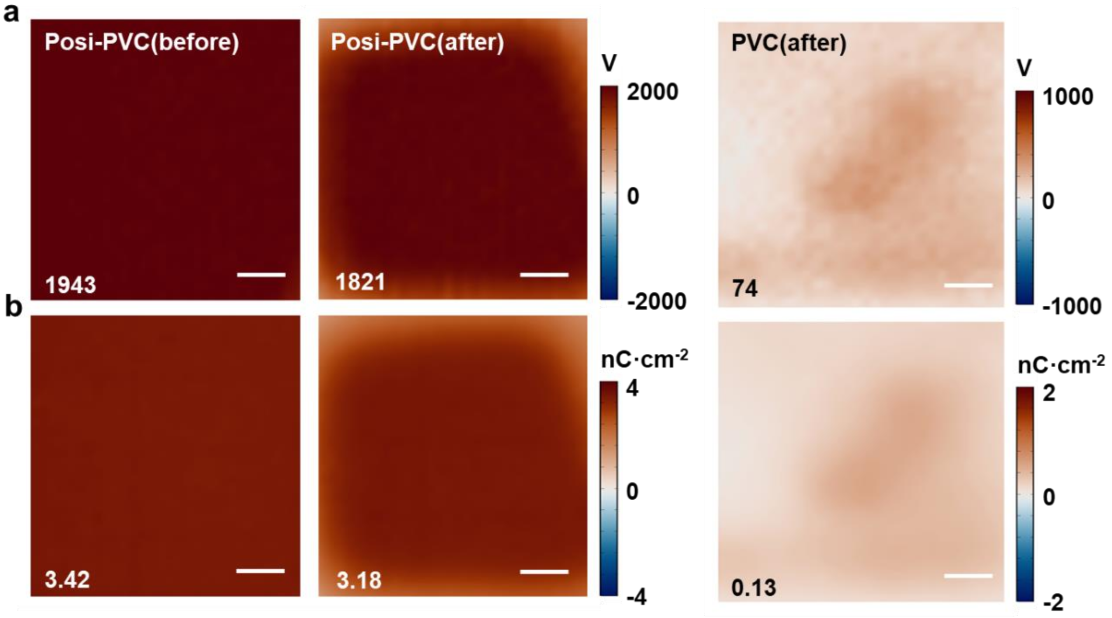

Supplementary Fig. 29 | The surface potential and surface charge distribution of the positive charged PVC based TENG before and after 5000 contact-separation cycles. a, Surface potential. **b**, Surface charge. (Scale bar: 3 mm)

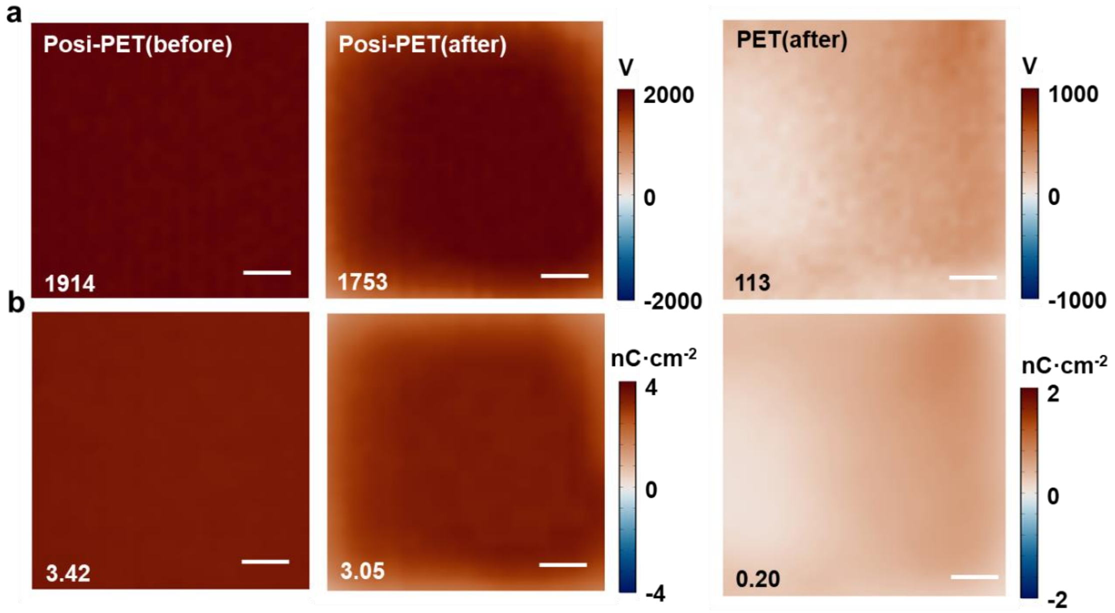

Supplementary Fig. 30 | The surface potential and surface charge distribution of the positive charged PET based TENG before and after 5000 contact-separation cycles. a, Surface potential. **b**, Surface charge. (Scale bar: 3 mm)

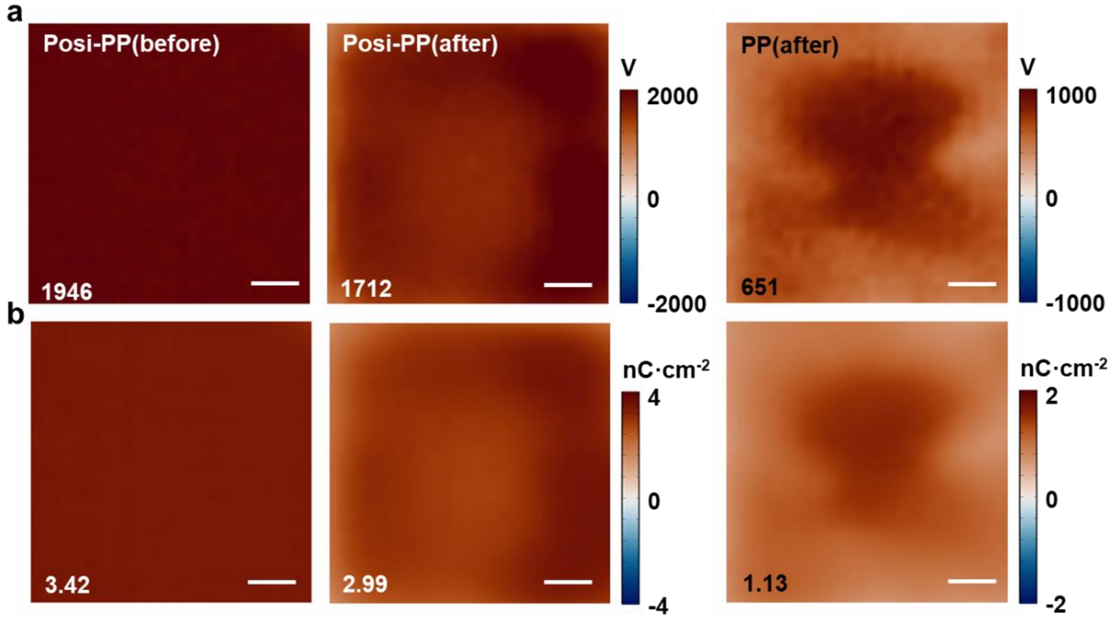

Supplementary Fig. 31 | The surface potential and surface charge distribution of the positive charged PP based TENG before and after 5000 contact-separation cycles. a, Surface potential. **b**, Surface charge. (Scale bar: 3 mm)

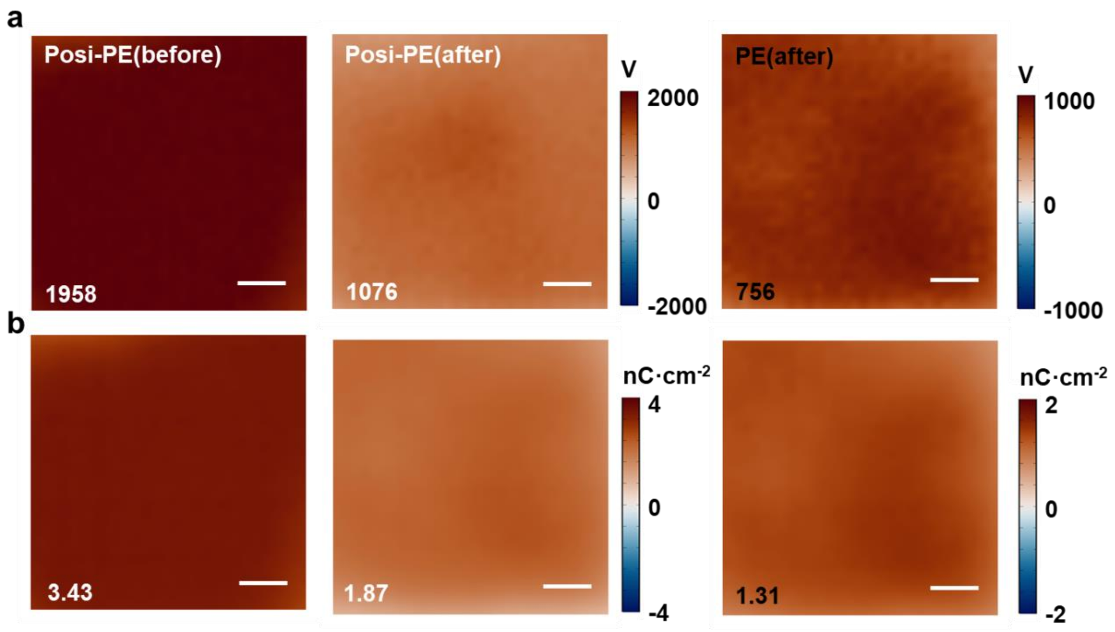

Supplementary Fig. 32 | The surface potential and surface charge distribution of the positive charged PE based TENG before and after 5000 contact-separation cycles. a, Surface potential. **b**, Surface charge. (Scale bar: 3 mm)

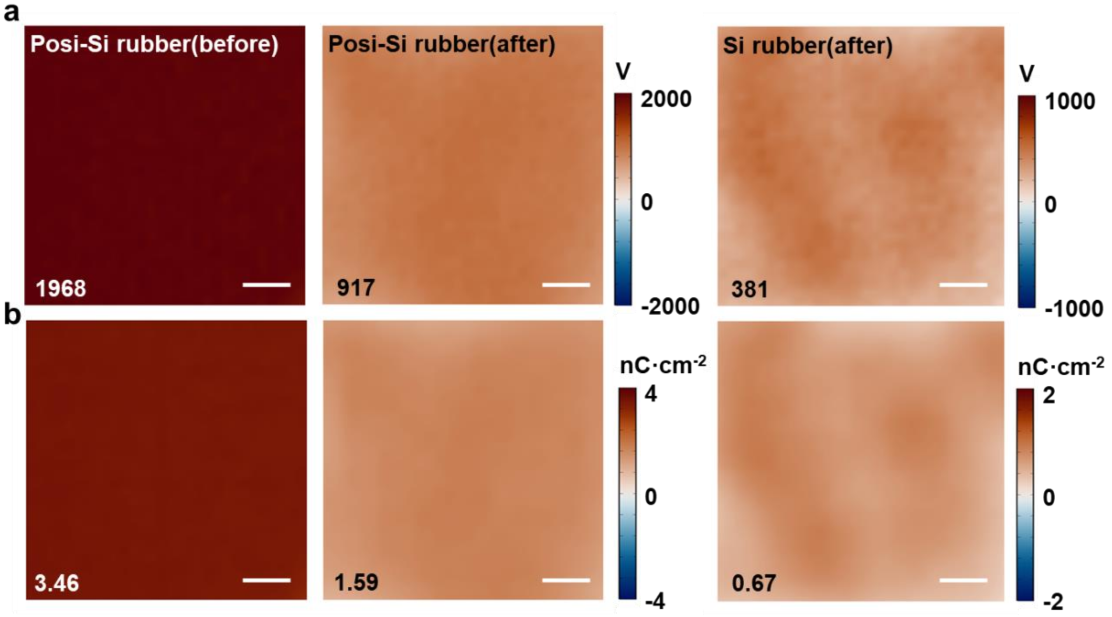

Supplementary Fig. 33 | The surface potential and surface charge distribution of the positive charged Si rubber based TENG before and after 5000 contact-separation cycles. **a**, Surface potential. **b**, Surface charge. (Scale bar: 3 mm)

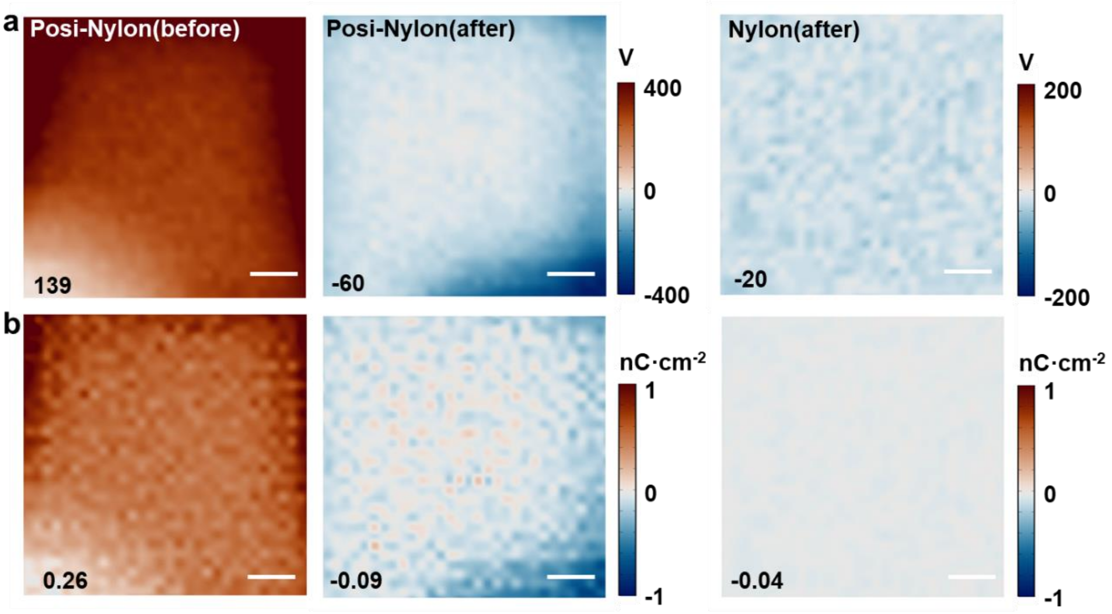

Supplementary Fig. 34 | The surface potential and surface charge distribution of the positive charged Nylon based TENG before and after 5000 contact-separation cycles. a, Surface potential. **b**, Surface charge. (Scale bar: 3 mm)

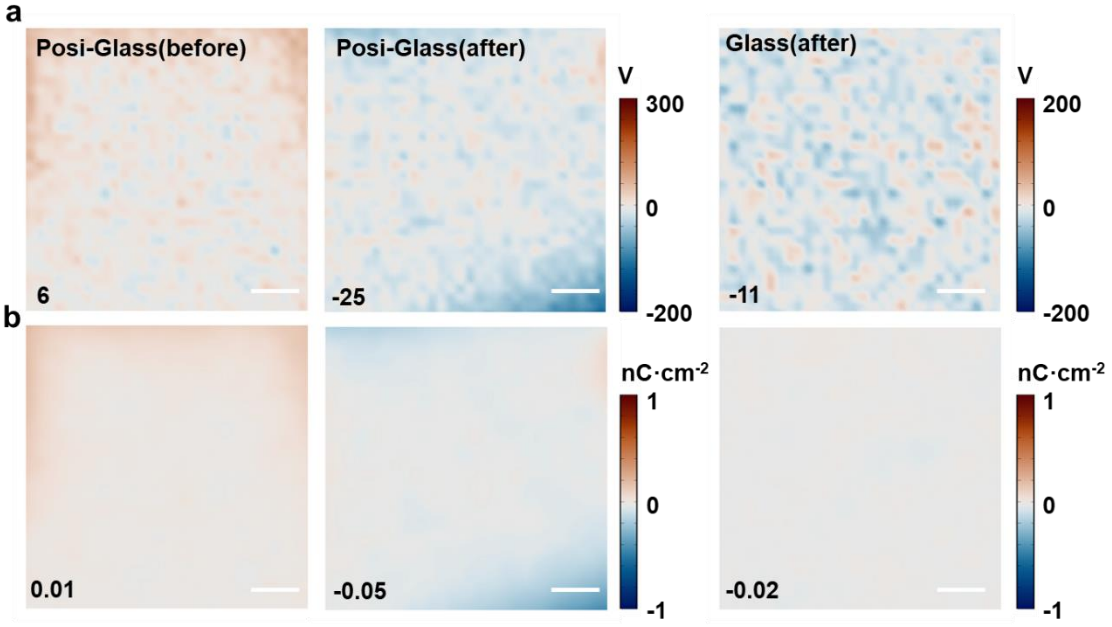

Supplementary Fig. 35 | The surface potential and surface charge distribution of the positive charged glass based TENG before and after 5000 contact-separation cycles. a, Surface potential. **b**, Surface charge. (Scale bar: 3 mm)

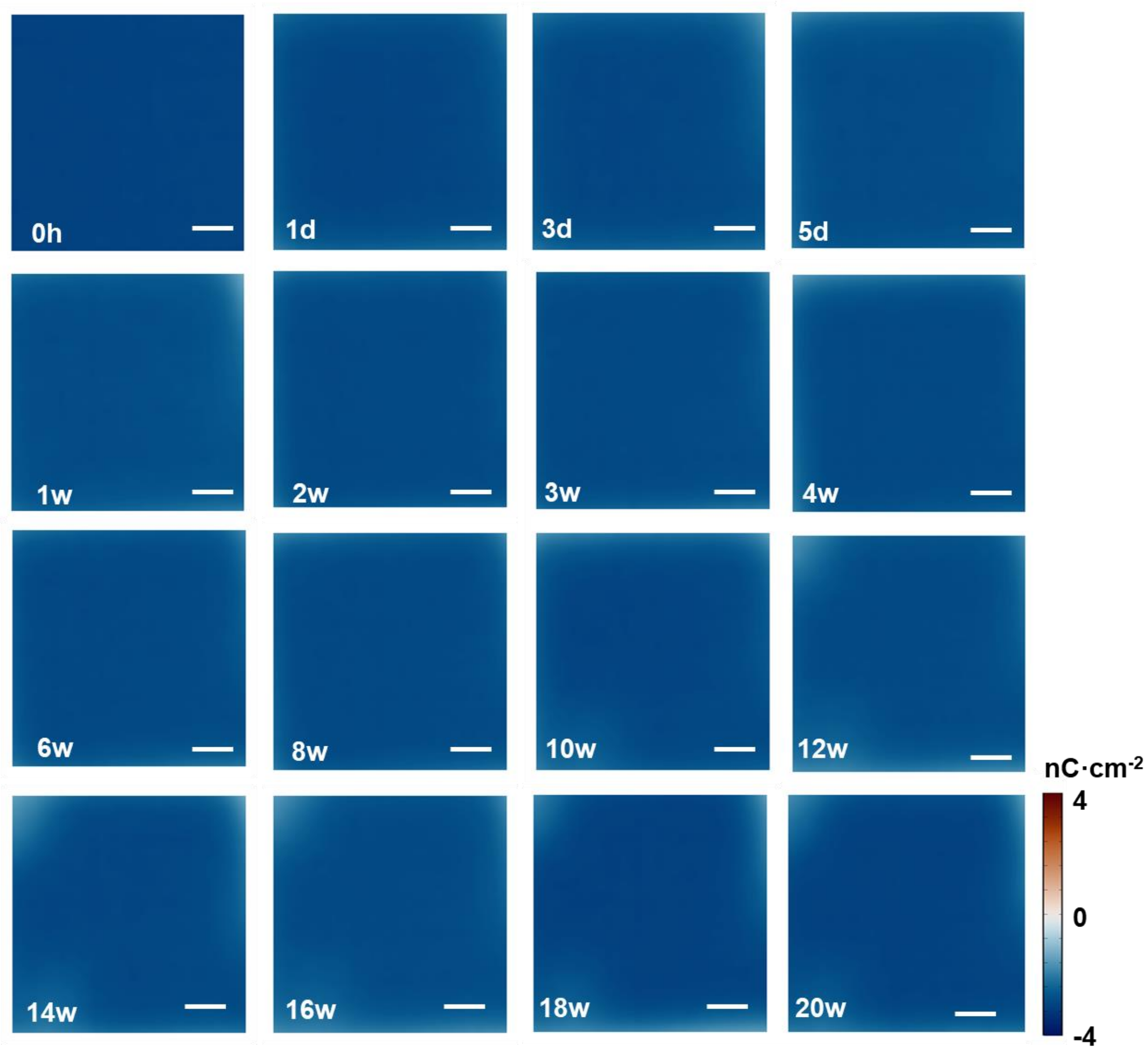

Supplementary Fig. 36 | Surface charge distribution of the neg-PTFE during dissipation. (Scale bar: 3 mm)

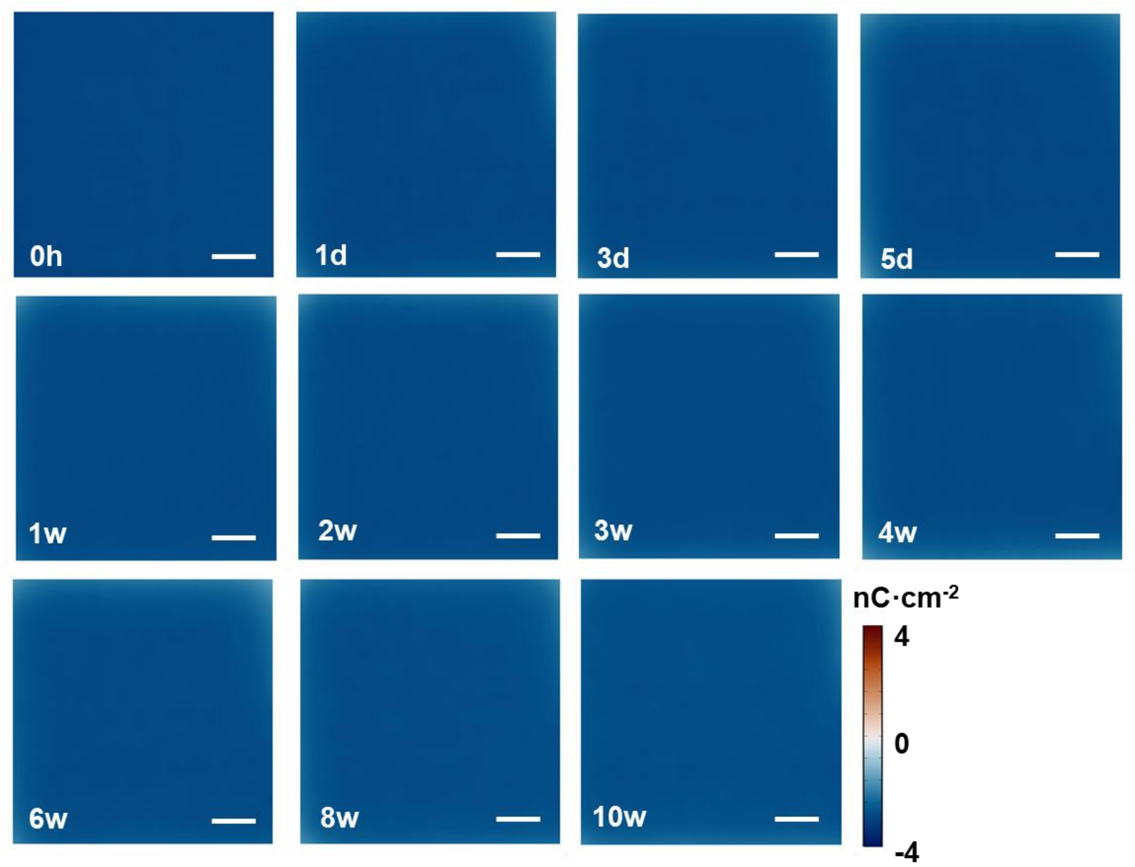

Supplementary Fig. 37 | Surface charge distribution of the neg-PFA during dissipation. (Scale bar: 3 mm)

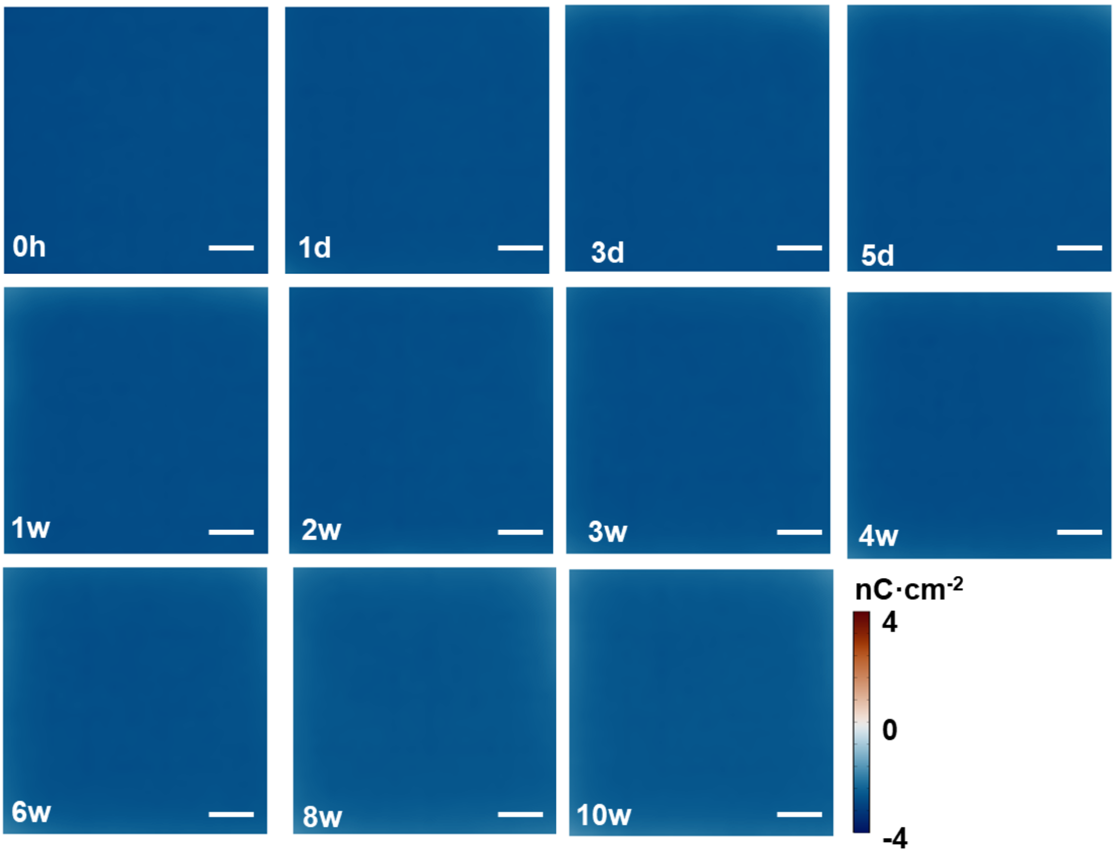

Supplementary Fig. 38 | Surface charge distribution of the neg-PVC during dissipation (Scale bar: 3 mm).

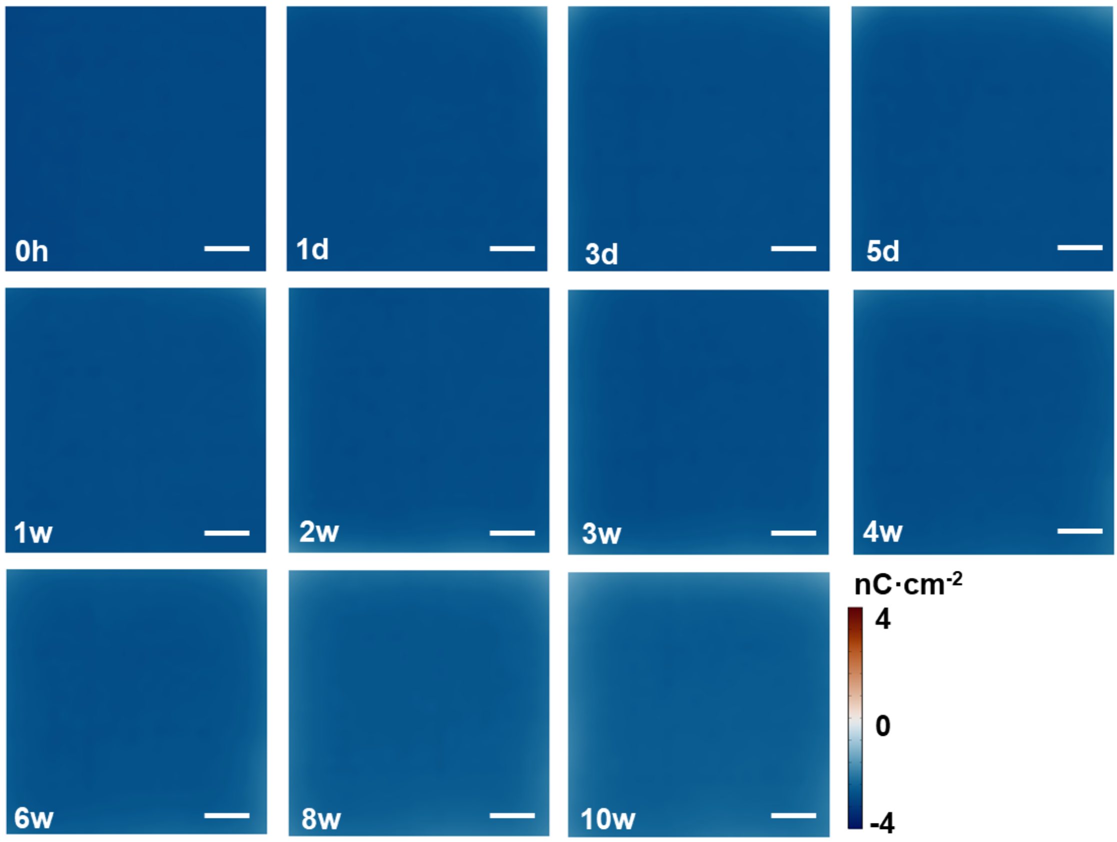

Supplementary Fig. 39 | Surface charge distribution of the neg-PET during dissipation (Scale bar: 3 mm).

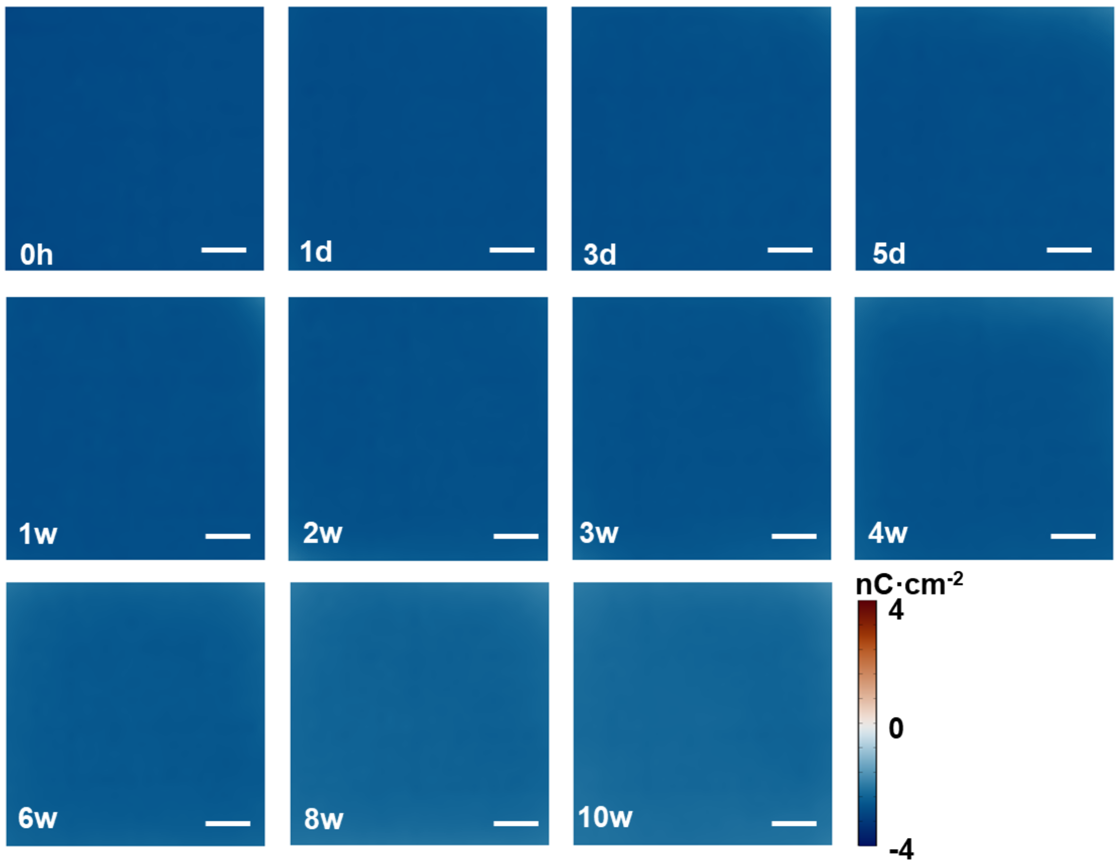

Supplementary Fig. 40 | Surface charge distribution of the neg-PP during dissipation (Scale bar: 3 mm).

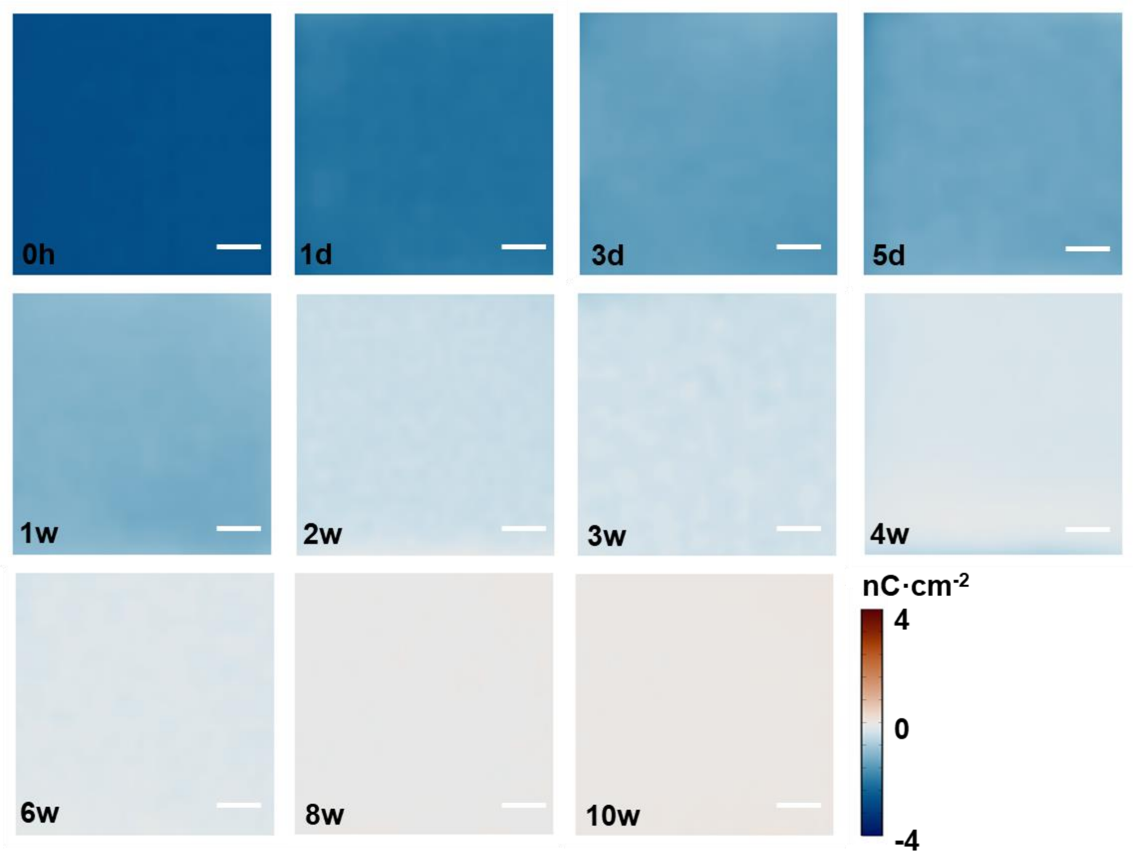

Supplementary Fig. 41 | Surface charge distribution of the neg-PE during dissipation (Scale bar: 3 mm).

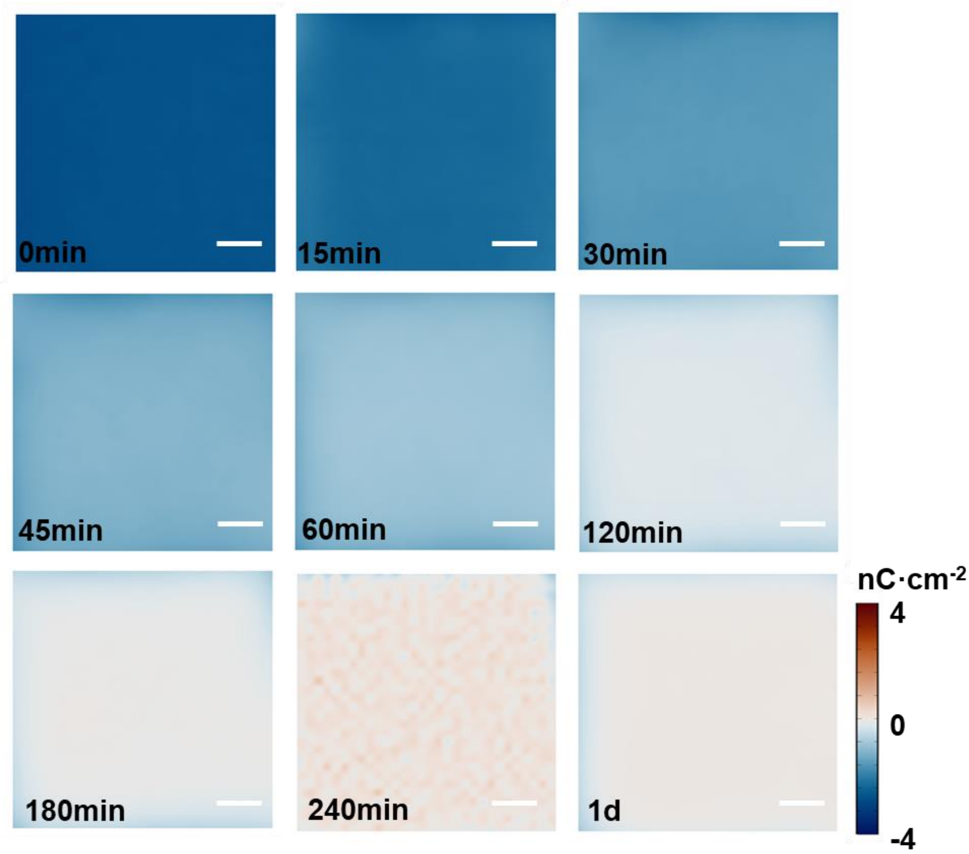

Supplementary Fig. 42 | Surface charge distribution of the neg-Si rubber during dissipation (Scale bar: 3 mm).

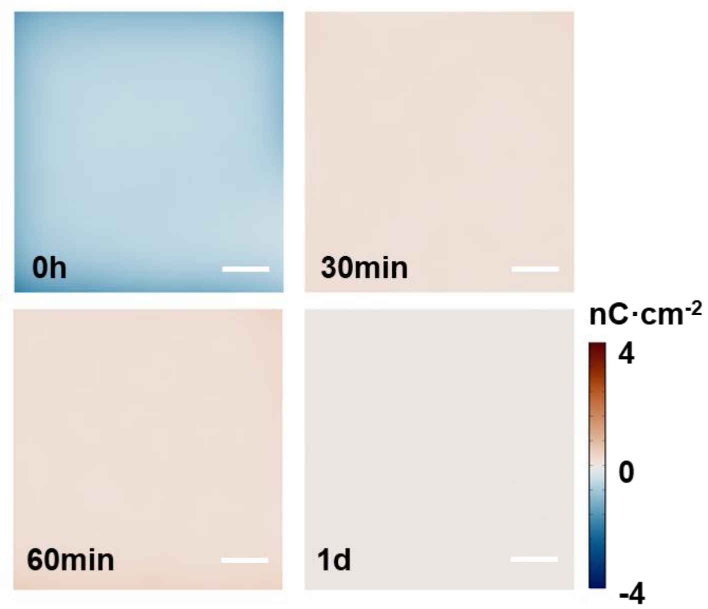

Supplementary Fig. 43 | Surface charge distribution of the neg-Nylon during dissipation (Scale bar: 3 mm).

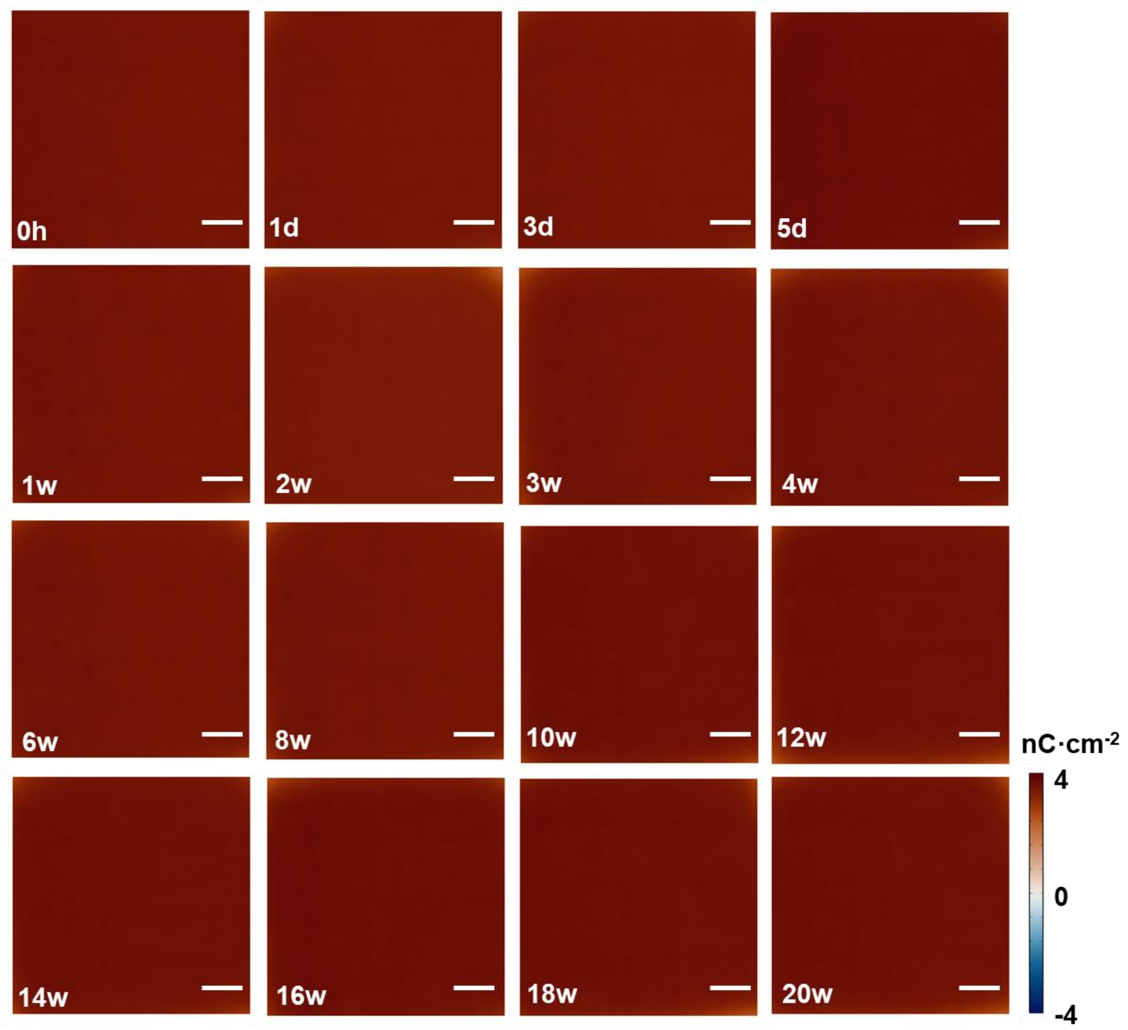

Supplementary Fig. 44 | Surface charge distribution of the posi-PTFE during dissipation (Scale bar: 3 mm).

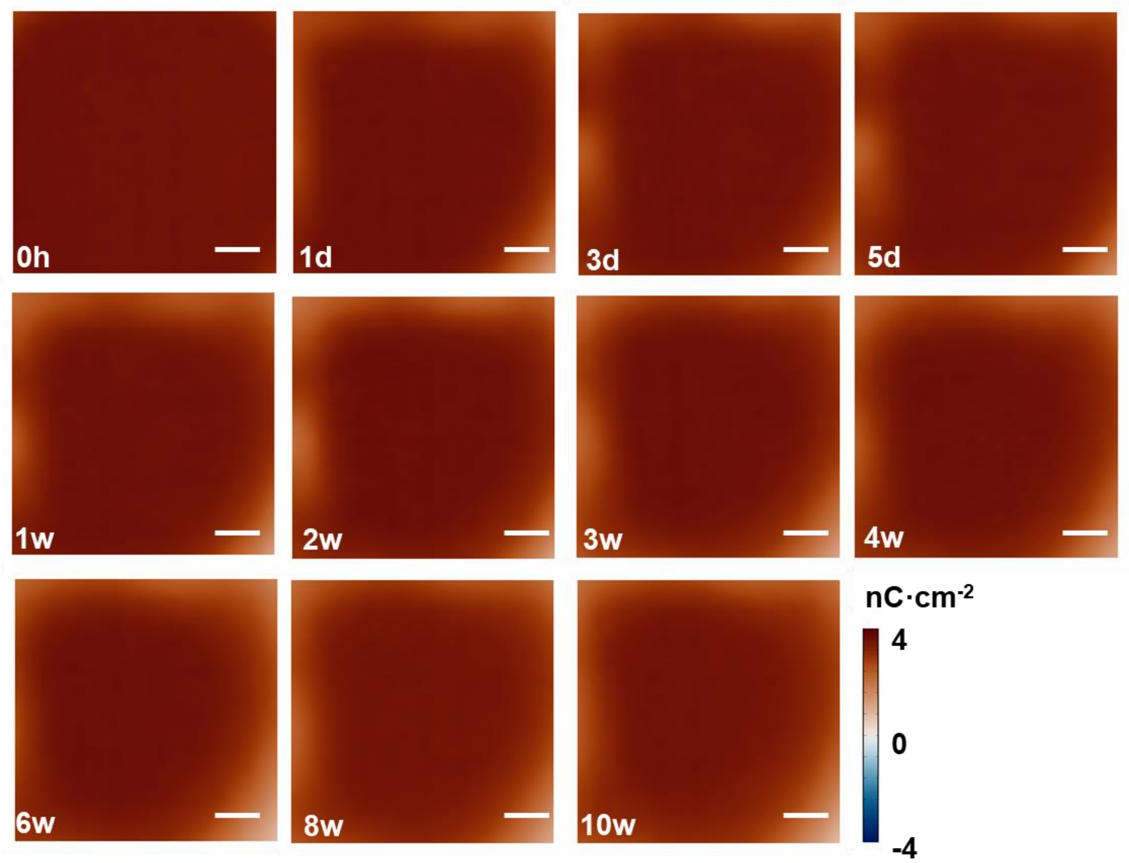

Supplementary Fig. 45 | Surface charge distribution of the posi-PFA during dissipation (Scale bar: 3 mm).

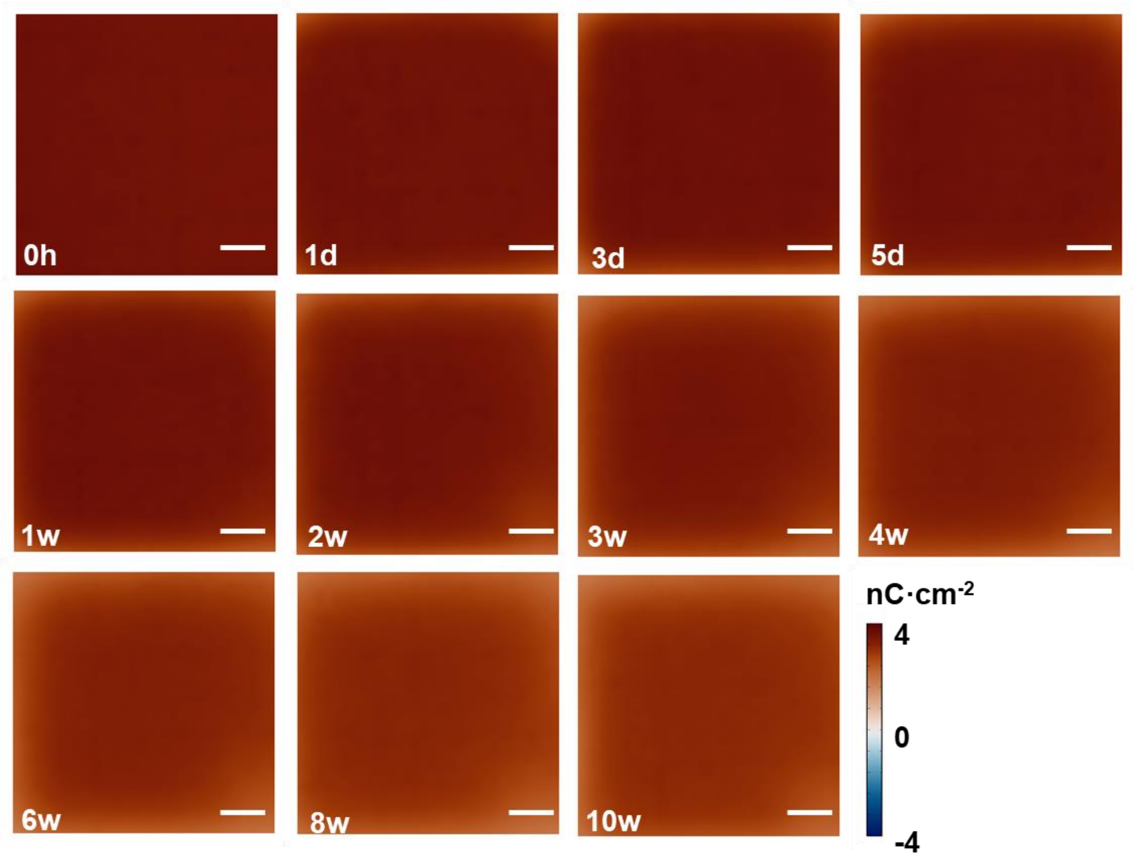

Supplementary Fig. 46 | Surface charge distribution of the posi-PVC during dissipation (Scale bar: 3 mm).

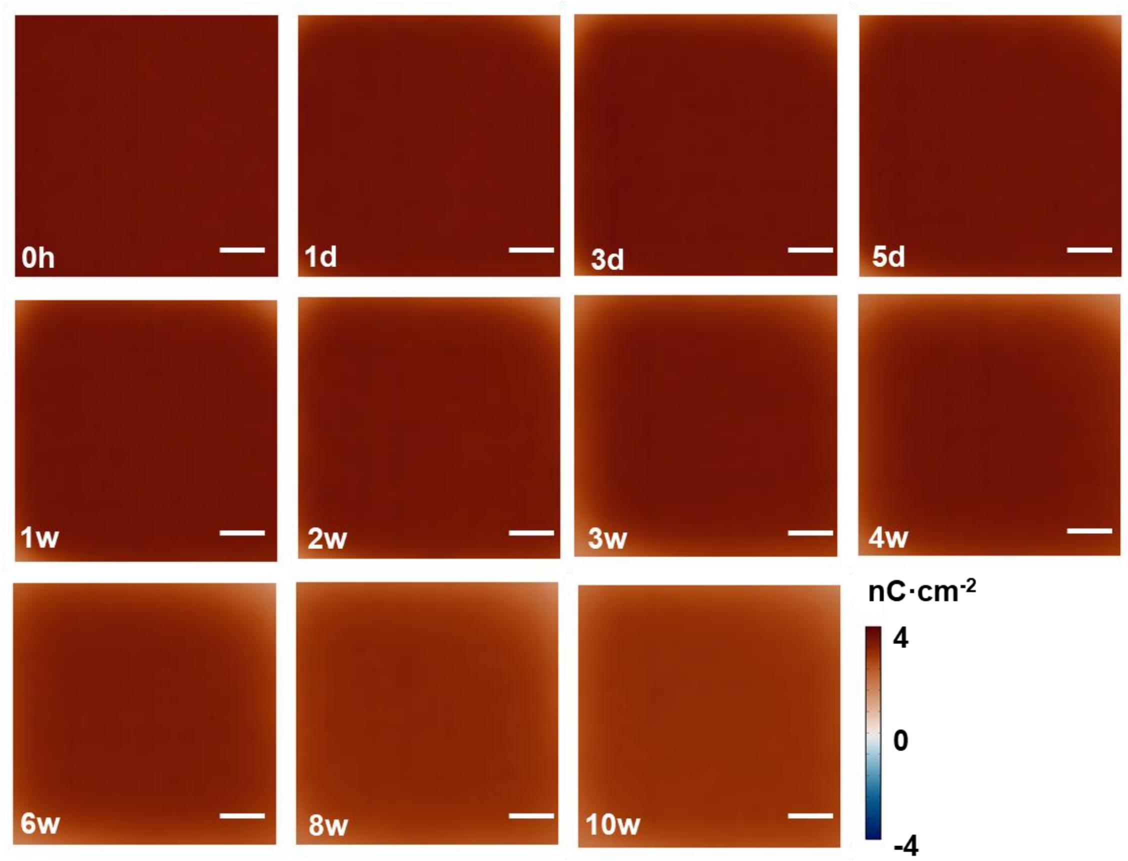

Supplementary Fig. 47 | Surface charge distribution of the posi-PET during dissipation (Scale bar: 3 mm).

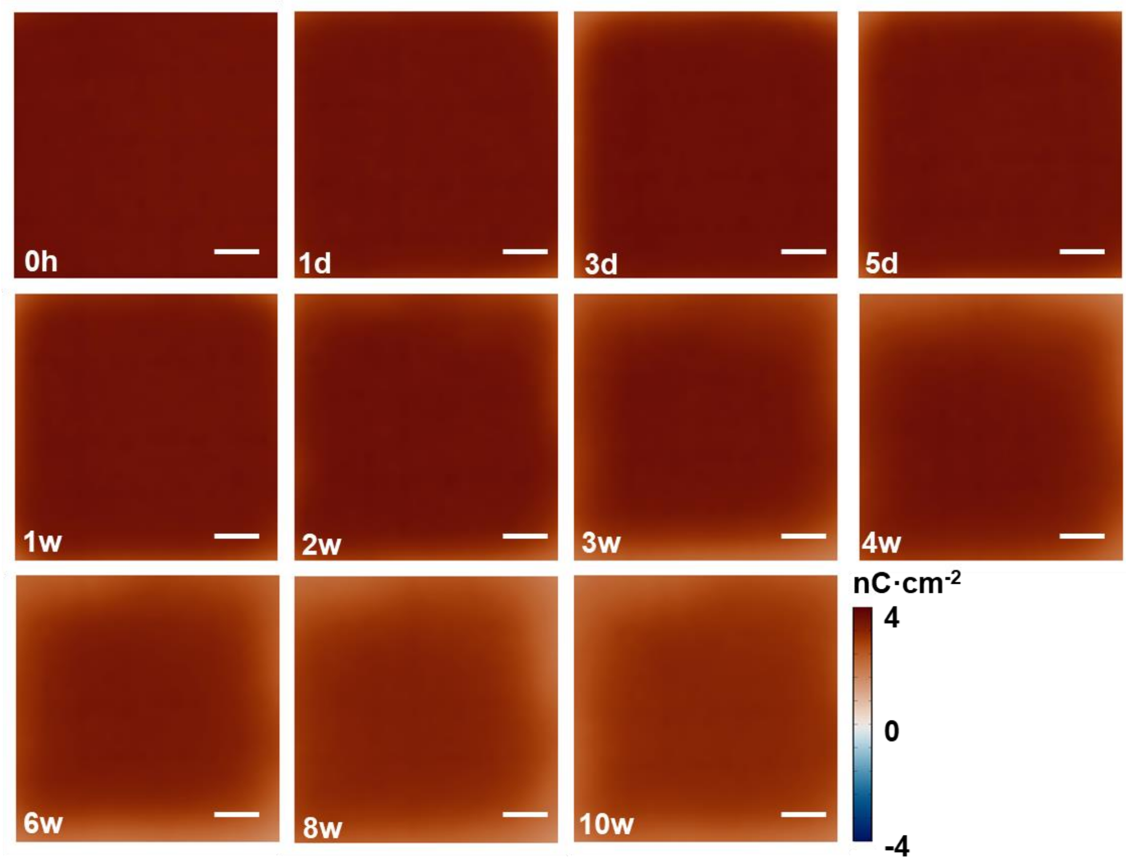

Supplementary Fig. 48 | Surface charge distribution of the posi-PP during dissipation (Scale bar: 3 mm).

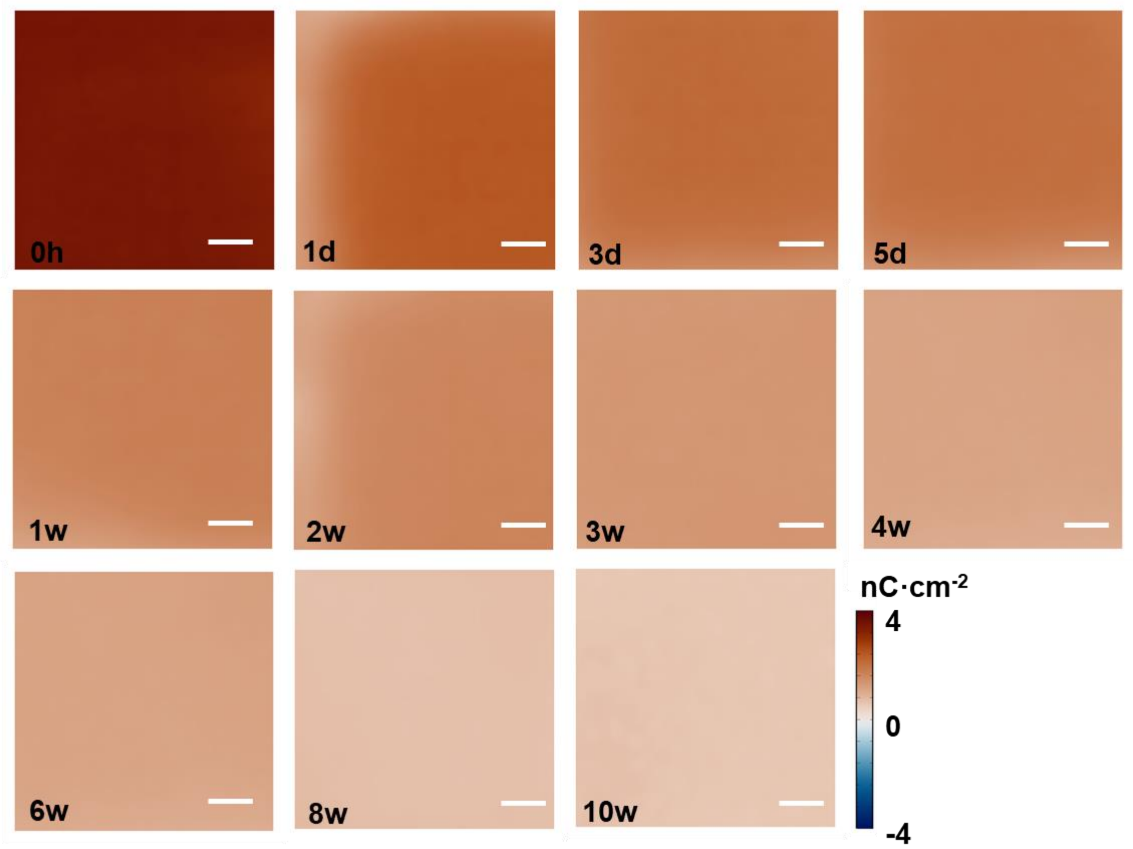

Supplementary Fig. 49 | Surface charge distribution of the posi-PE during dissipation (Scale bar: 3 mm).

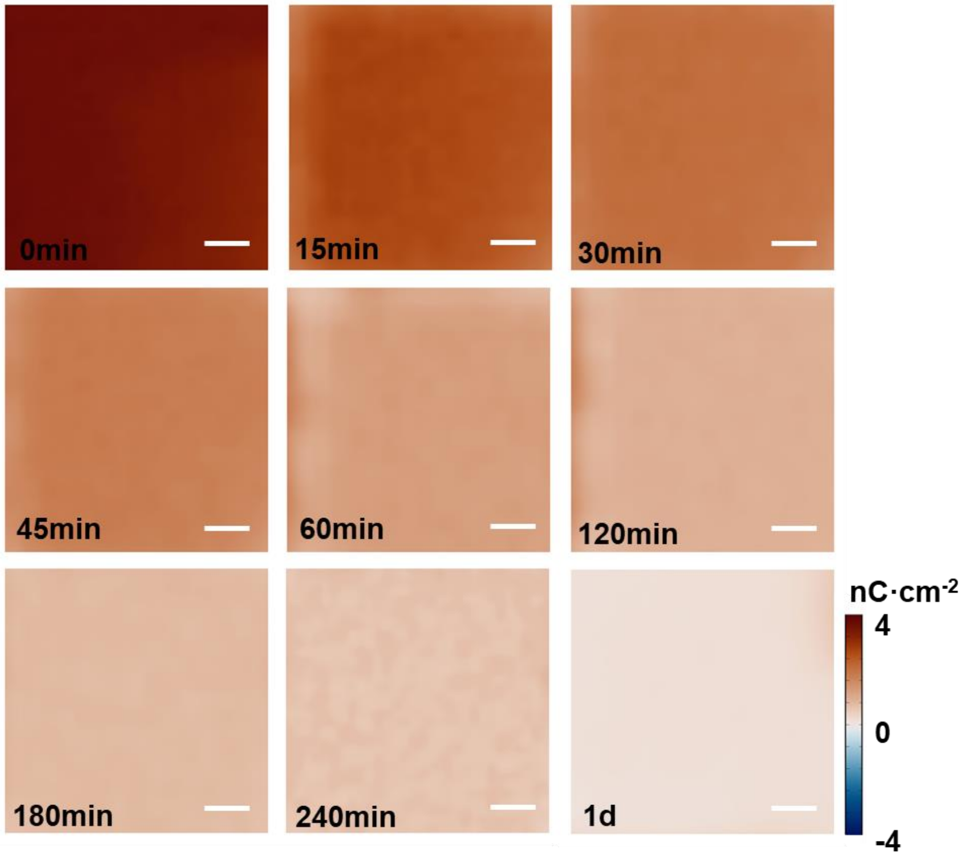

Supplementary Fig. 50 | Surface charge distribution of the posi-Si rubber during dissipation (Scale bar: 3 mm).

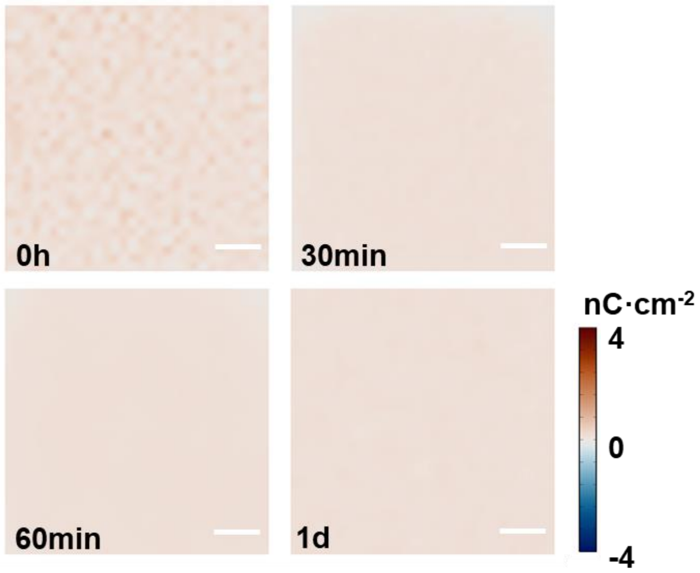

Supplementary Fig. 51 | Surface charge distribution of the posi-Nylon during dissipation (Scale bar: 3 mm).

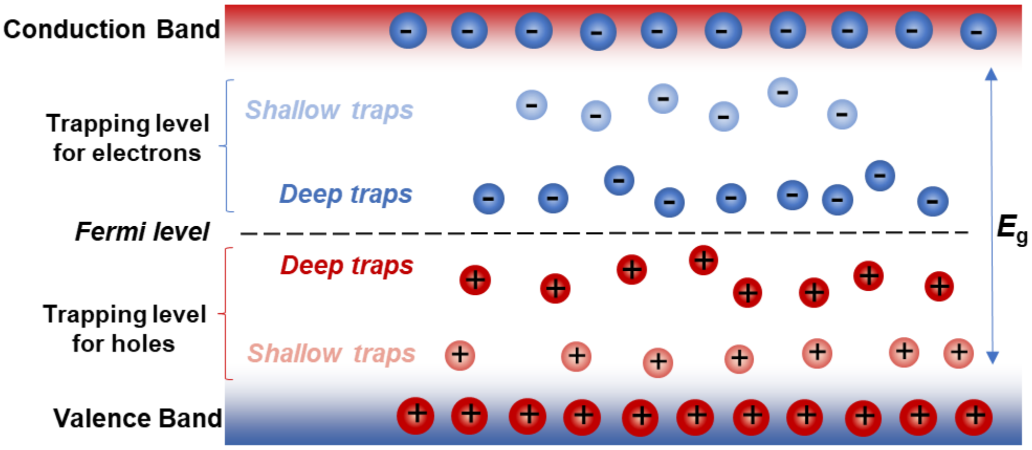

Supplementary Fig. 52 | Schematic diagram of the distribution of shallow and deep traps for electrons and holes in tribo-dielectric material.

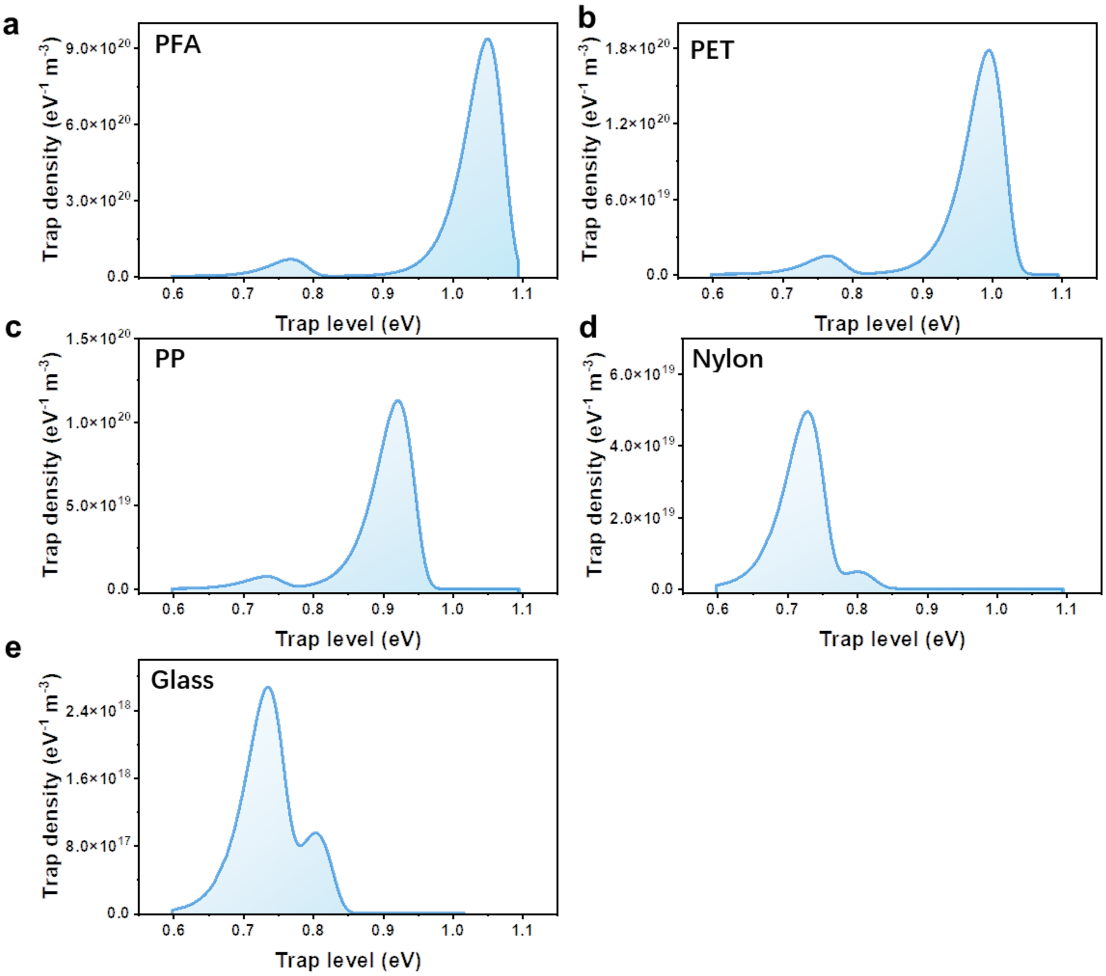

Supplementary Fig. 53 | Electron trap distribution of polymers. **a**, PFA. **b**, PET. **c**, PP. **d**, Nylon. **e**, Glass.

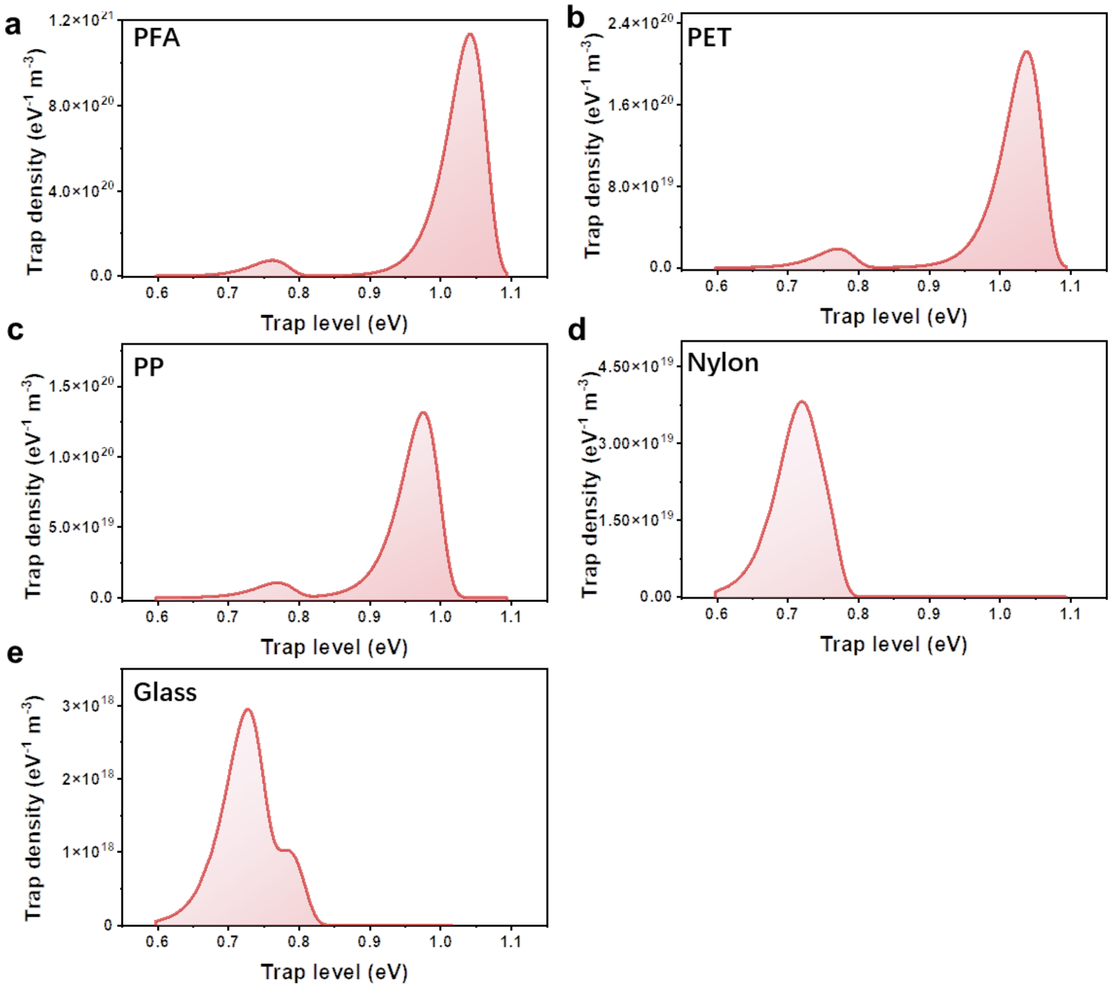

Supplementary Fig. 54 | Hole trap distribution of polymers. **a**, PFA. **b**, PET. **c**, PP. **d**, Nylon. **e**, Glass.

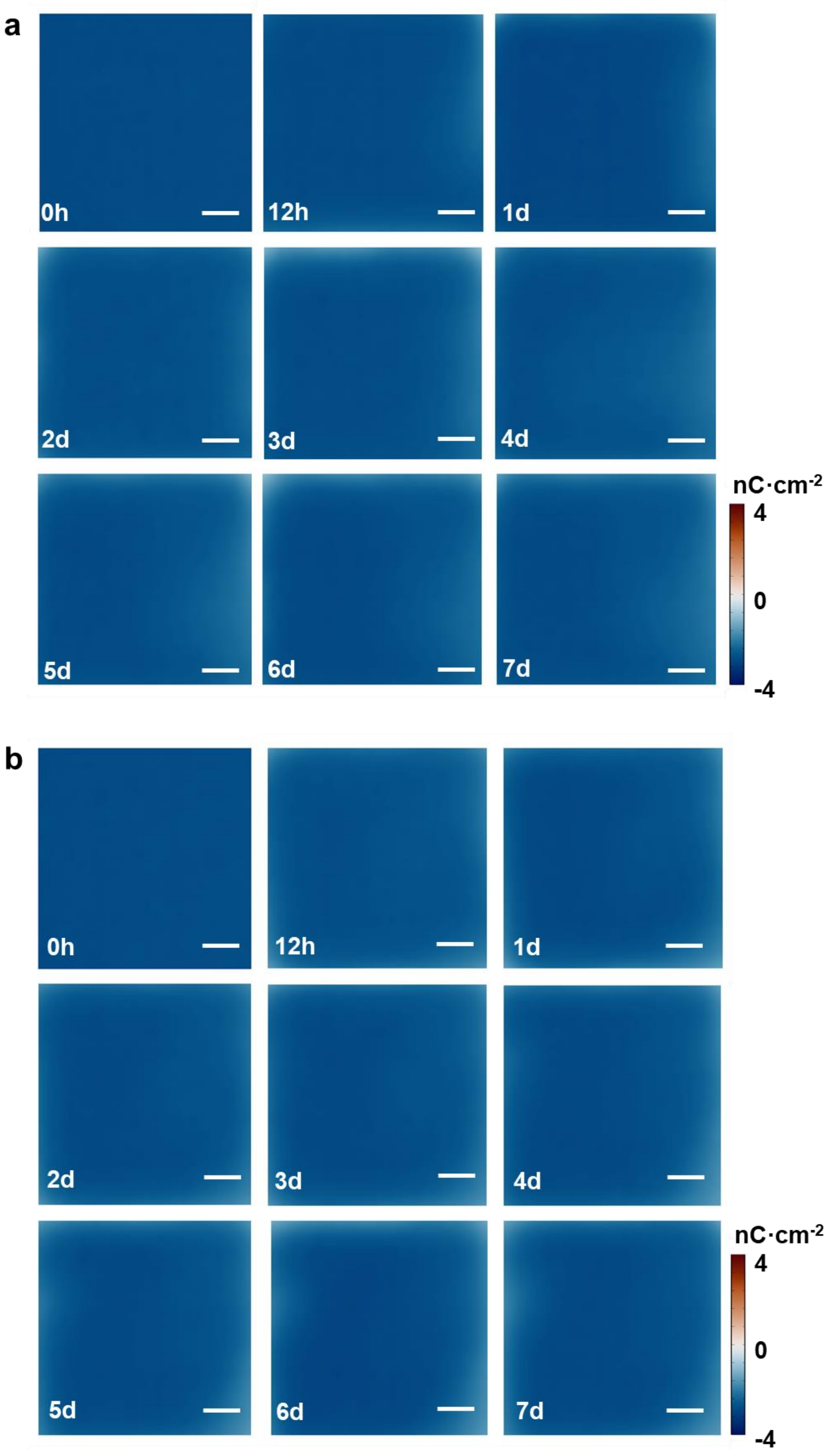

a
0h
12h
1d
2d
3d
4d
5d
6d
7d
nC·cm-2
4
0
-4
b
0h
12h
1d
2d
3d
4d
5d
6d
7d
nC·cm-2
4
0
-4

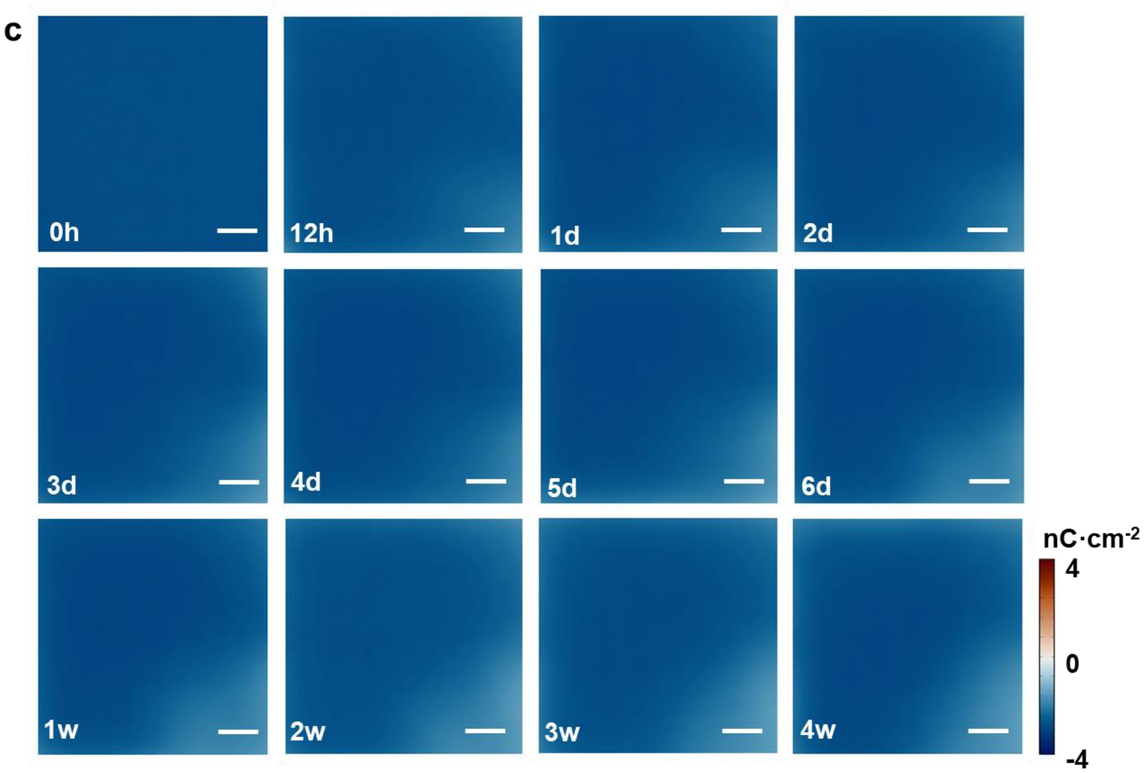

Supplementary Fig. 55 | Surface charge distribution of the neg-PTFE during dissipation under different relative humidity conditions. a, 50% R.H. **b**, 70% R.H. **c**, 90% R.H. (Scale bar: 3 mm)

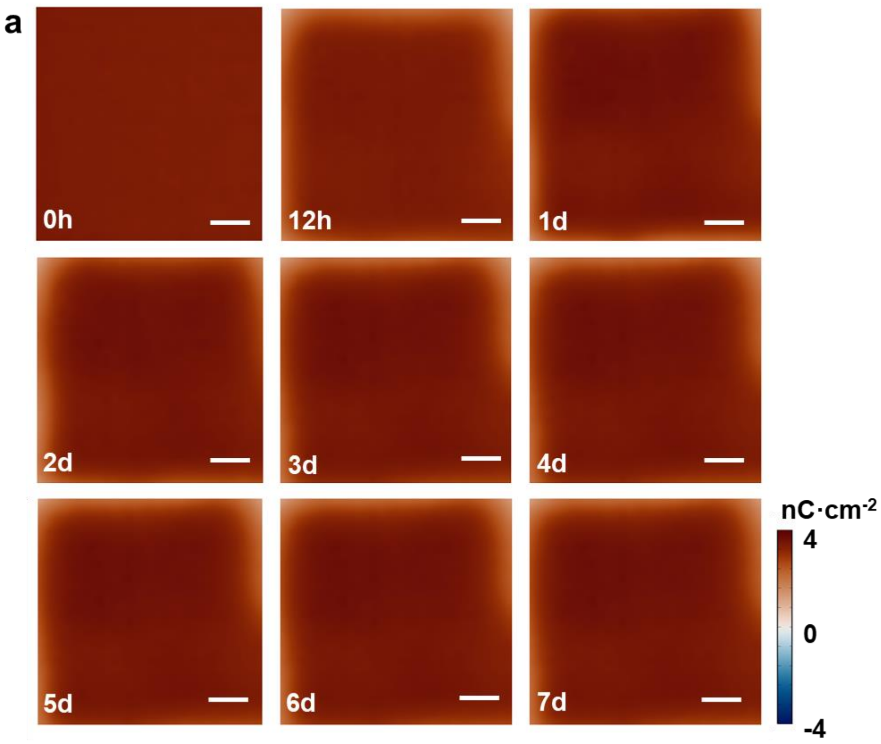
a
0h
12h
1d
2d
3d
4d
5d
6d
7d
nC·cm-2
4
0
-4

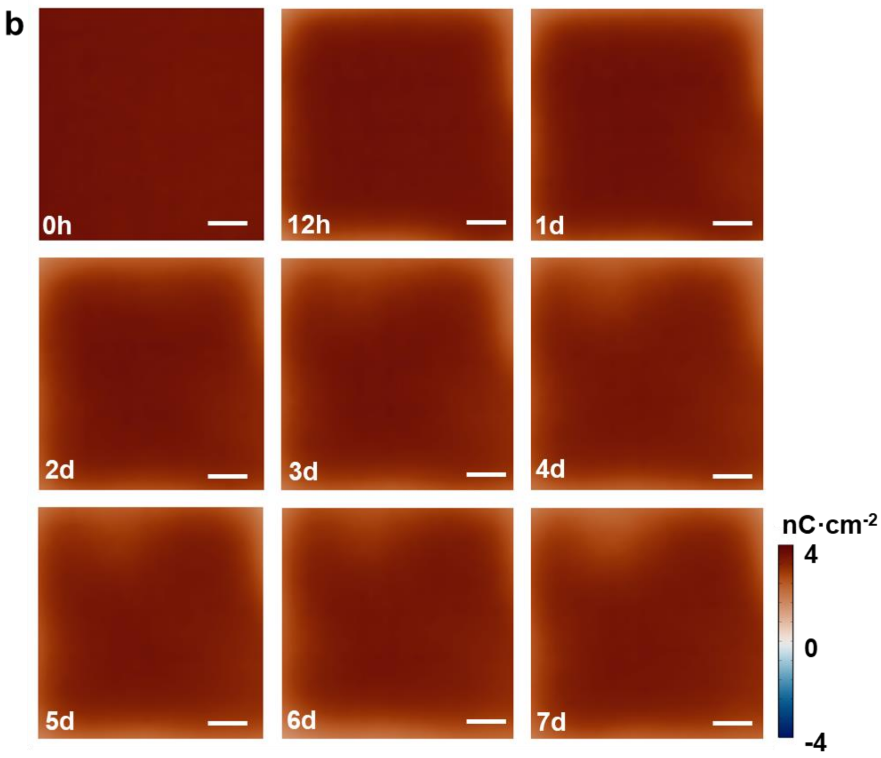
b
0h
12h
1d
2d
3d
4d
5d
6d
7d
nC·cm-2
4
0
-4

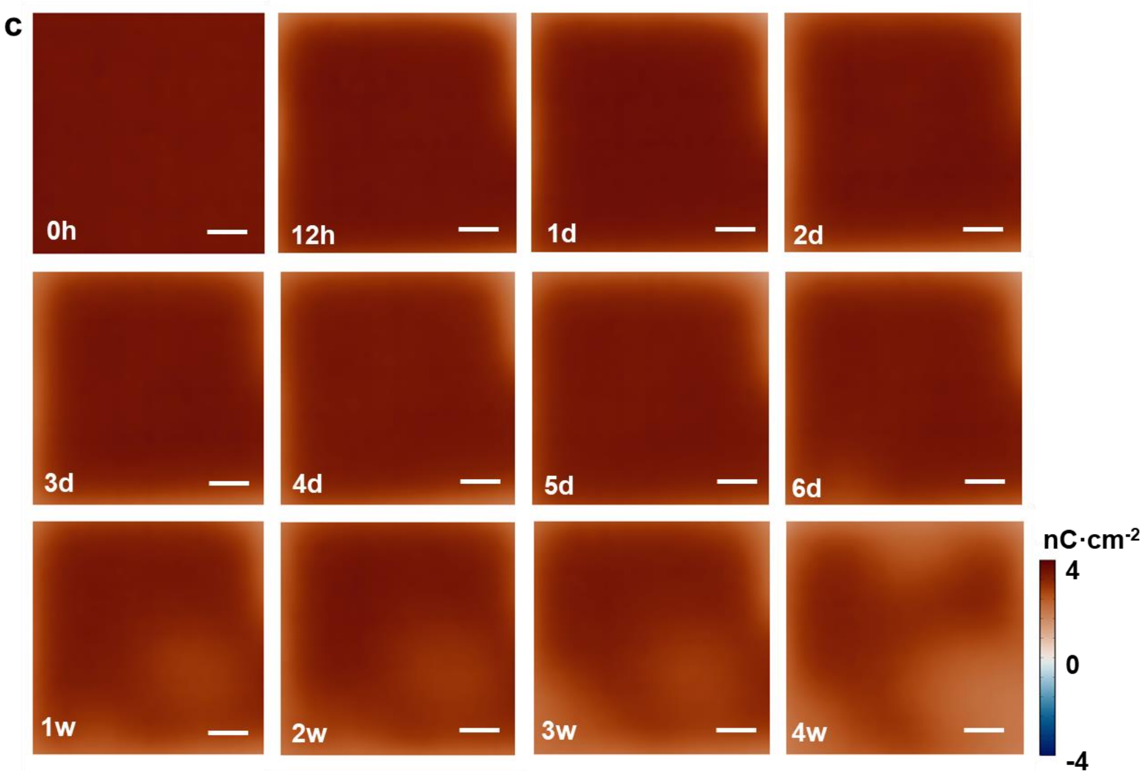

Supplementary Fig. 56 | Surface charge distribution of the posi-PTFE during dissipation under different relative humidity conditions. a, 50% R.H. **b**, 70% R.H. **c**, 90% R.H. (Scale bar: 3 mm)

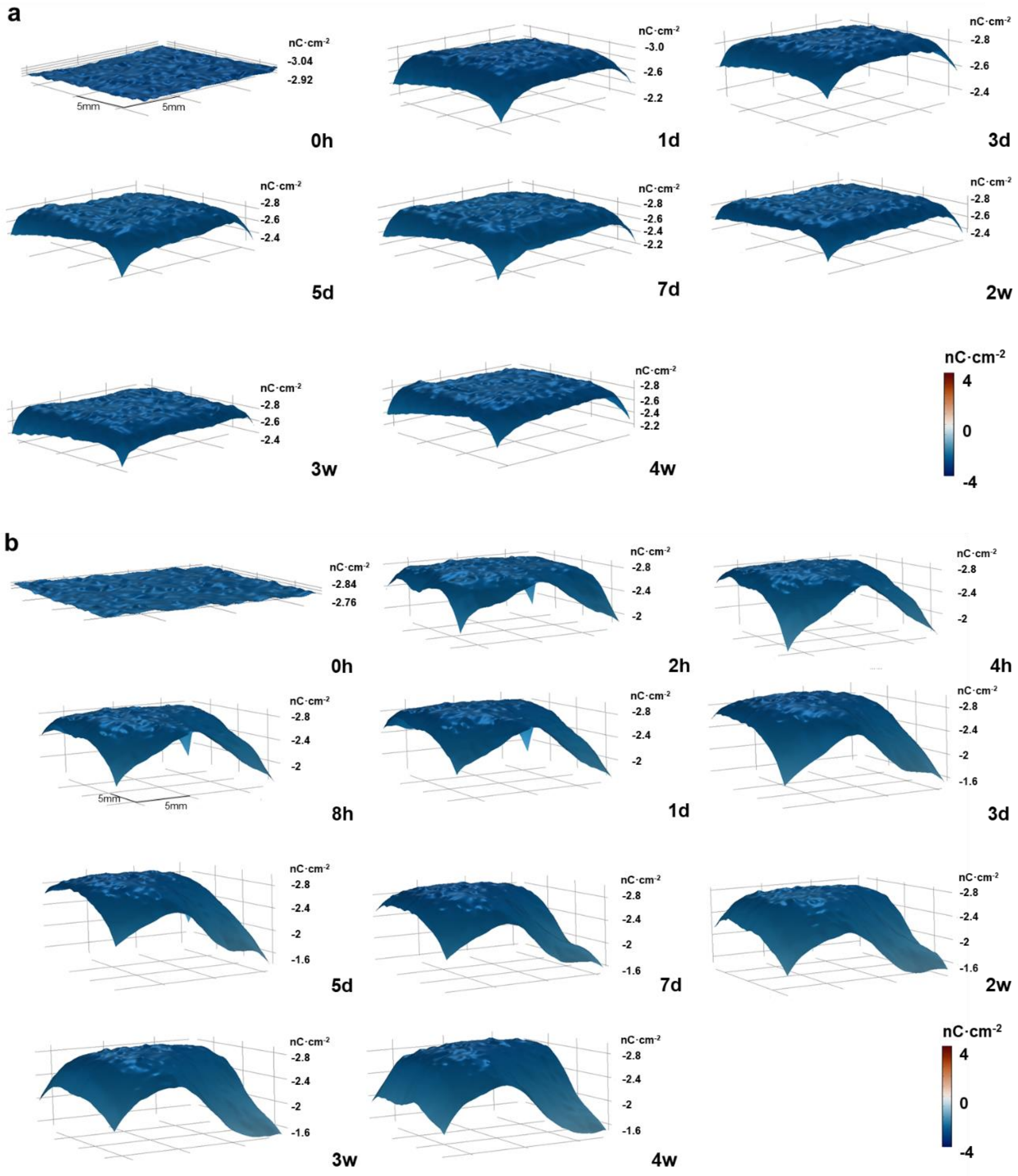

Supplementary Fig. 57 | The 3D negative surface charge distribution of the PTFE during long-term dissipation. a, 30% R.H. **b**, 90% R.H.

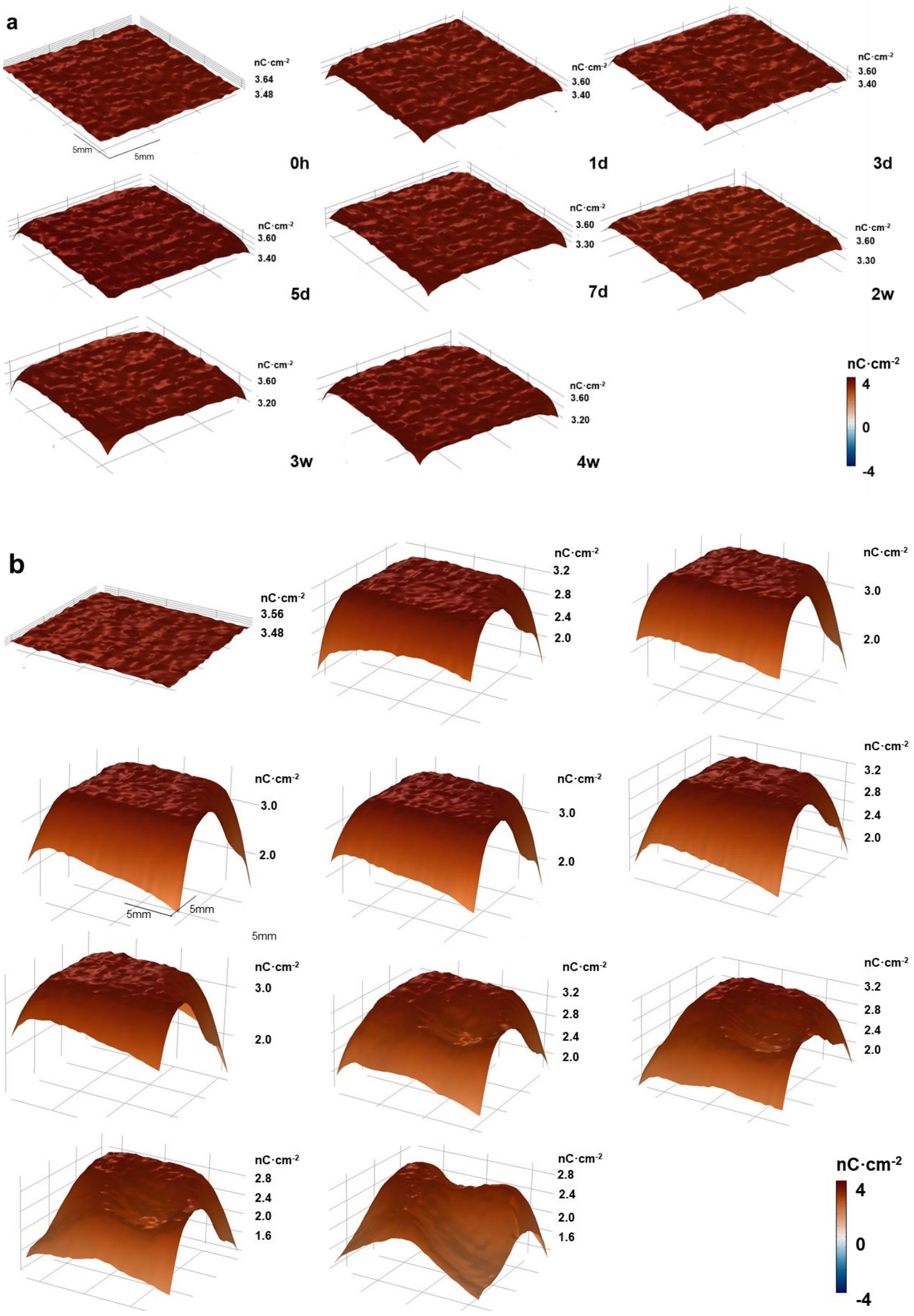

Supplementary Fig. 58 | The 3D positive surface charge distribution of the PTFE during long-term dissipation. **a**, 30% R.H. **b**, 90% R.H.

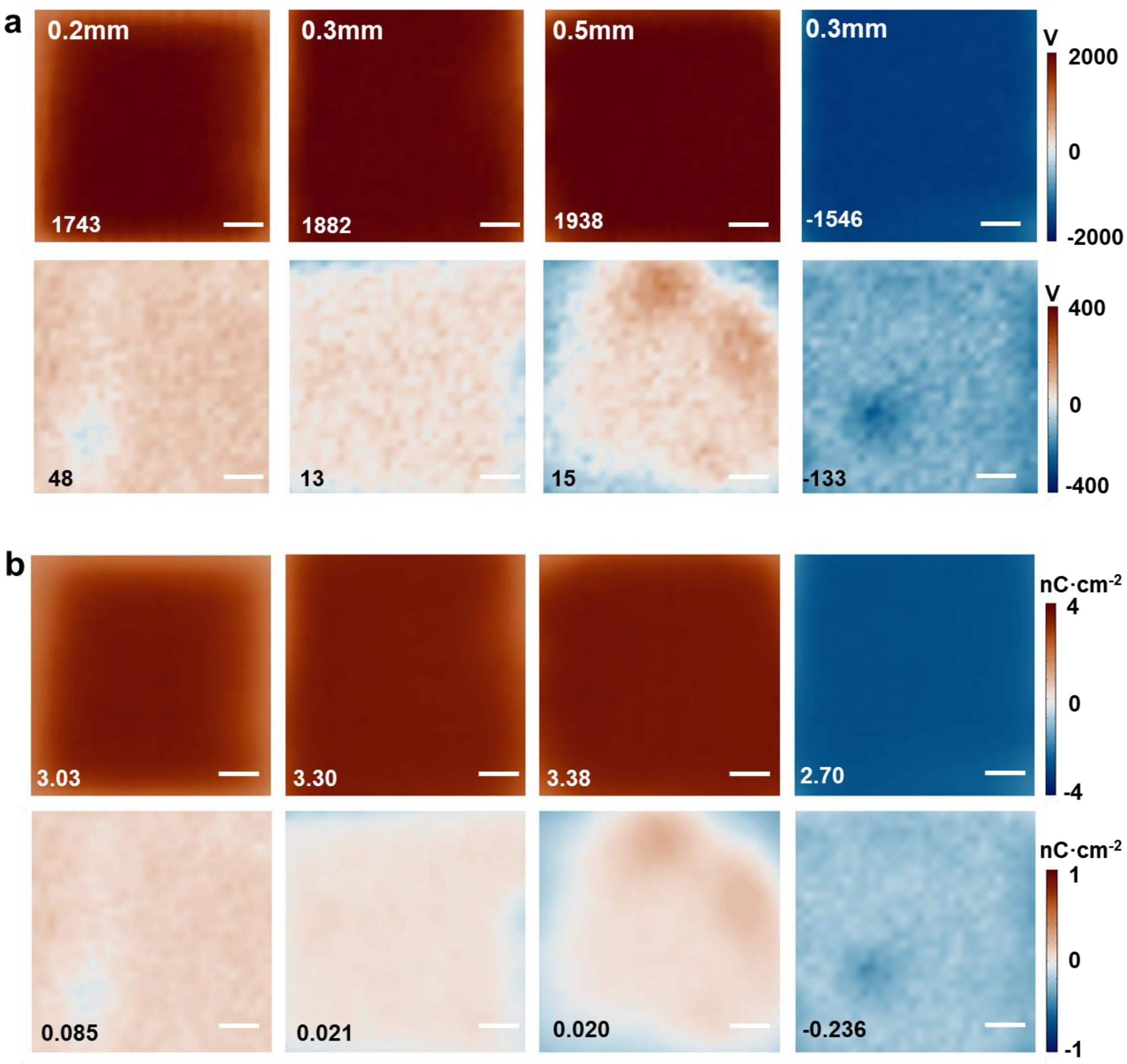

Supplementary Fig. 59 | The surface potential and surface charge distribution of the posi-PTFE or neg-PTFE based non-contact TENG at different gap distances. a, Surface potential **b**, Surface charge (Scale bar: 3 mm).

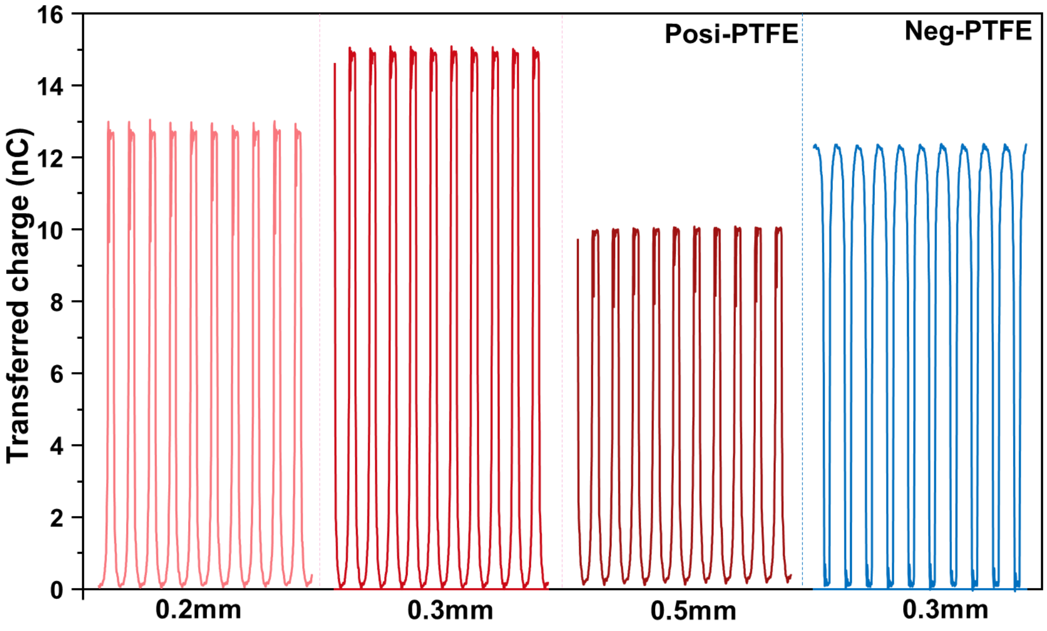

Supplementary Fig. 60 | The transferred charge waveform of the posi-PTFE or neg-PTFE based non-contact TENG at different gap distances.

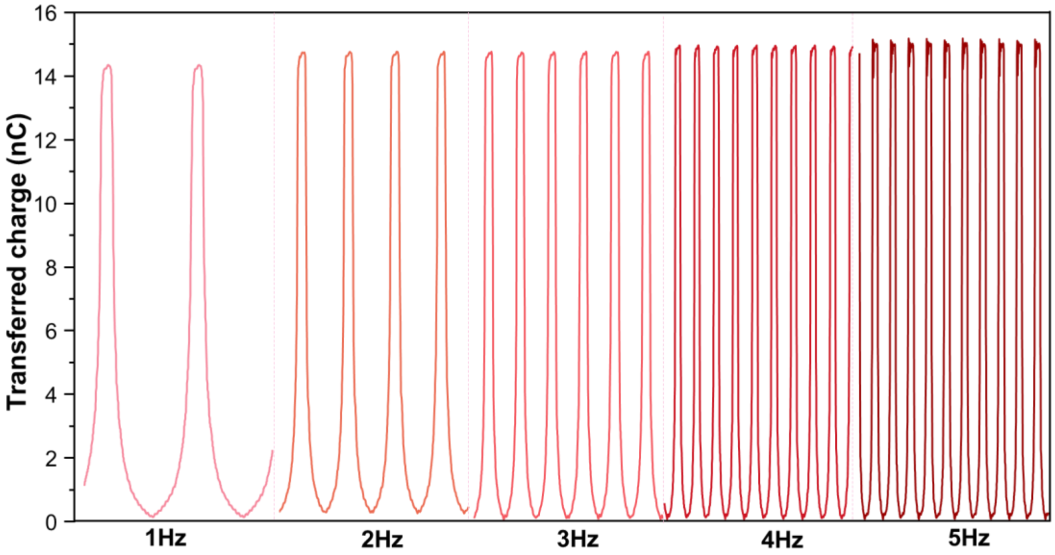

Supplementary Fig. 61 | The transferred charge waveform of the posi-PTFE based non-contact TENG at different approach-separation frequencies.

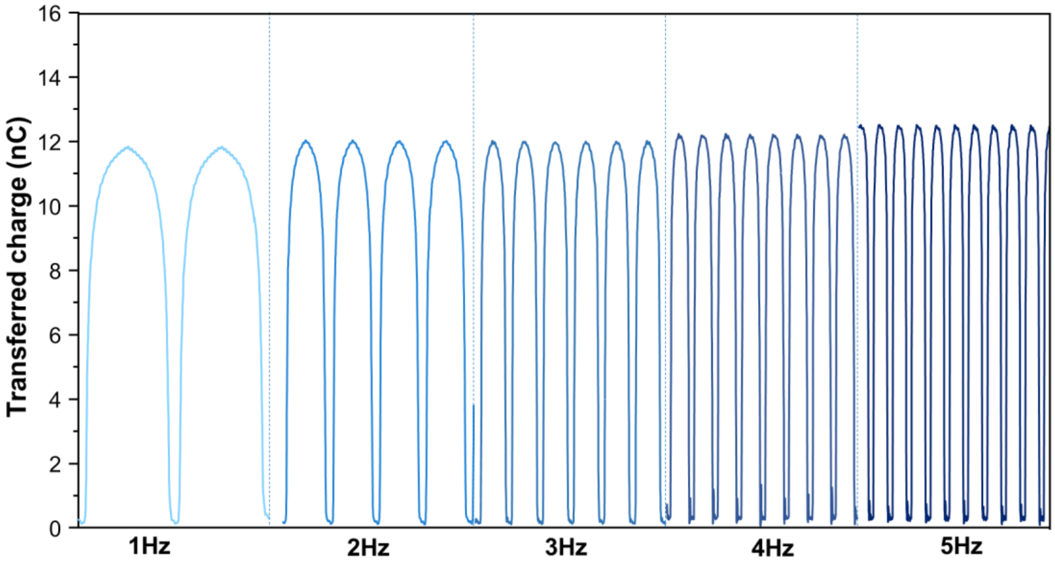

Supplementary Fig. 62 | The transferred charge waveform of the neg-PTFE based non-contact TENG at different approach-separation frequencies.

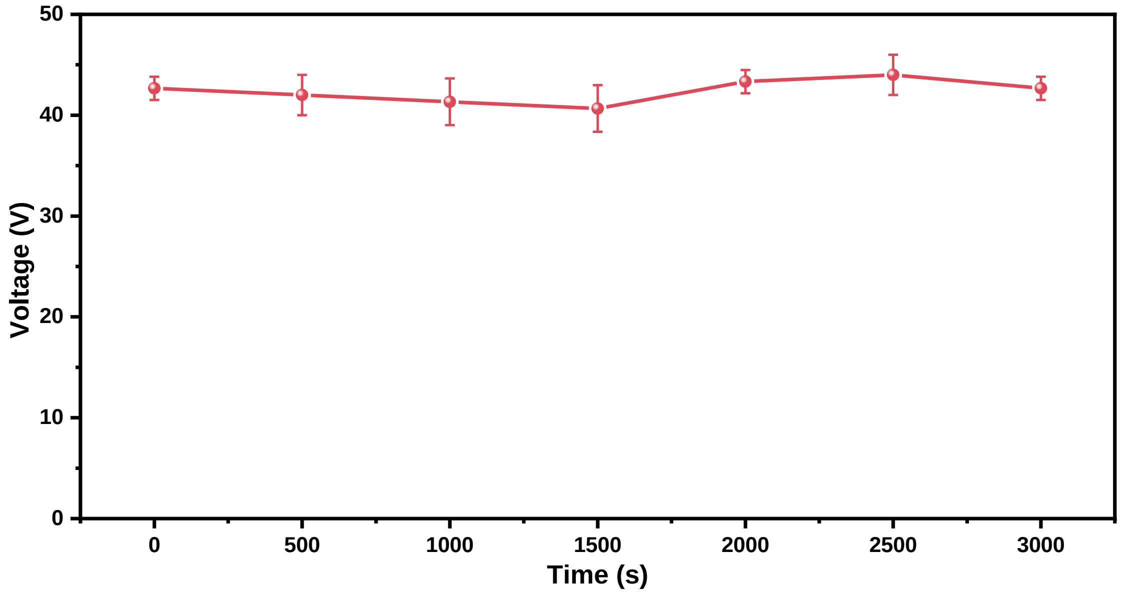

Supplementary Fig. 63 | The long-term output voltage durability of the posi-PTFE based non-contact TENG. (Distinct samples, mean ± s.d., $n = 5$)

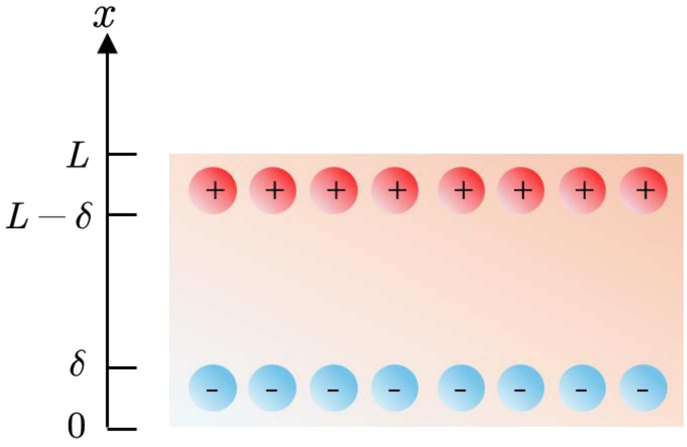

Supplementary Fig. 64 | Schematic diagram of charge distribution in the sample surface after positive polarity corona charge.

Supplementary References

[1] Kumada, A., Okabe, S. Charge distribution measurement on a truncated cone spacer under DC voltage. *IEEE Transactions on Dielectrics and Electrical Insulation*, ***11***, 929-938 (2004).

[2] Zhang B, Gao W, Qi Z, Wang Q, Zhang G. Inversion algorithm to calculate charge density on solid dielectric surface based on surface potential measurement. *IEEE Transactions on Instrumentation and Measurement*, ***66***, 3316-3326 (2017).

[3] Donatelli M, Ferrari P, Gazzola S. Symmetrization techniques in image deblurring. *arXiv preprint arXiv:**2212***, 05879 (2022).

[4] Chung J, Gazzola S. Flexible Krylov methods for L_p regularization. *SIAM Journal on Scientific Computing*, ***41***, 149-171 (2019).

[5] Golub G H, Heath M, Wahba G. Generalized cross-validation as a method for choosing a good ridge parameter. *Technometrics*, ***21***, 215-223 (1979).

[6] Gan M, Zhu H T, Chen G Y, Chen C P. Weighted generalized cross-validation-based regularization for broad learning system. *IEEE Transactions on Cybernetics*, ***52***, 4064-4072 (2020).

[7] Kumada A, Okabe S, Hidaka K. Residual charge distribution of positive surface streamer. *Journal of Physics D: Applied Physics*, ***42***, 095209 (2009).

[8] Apodaca, M. M., Wesson, P. J., Bishop, K. J., Ratner, M. A., & Grzybowski, B. A. (2010). Contact electrification between identical materials. *Angewandte Chemie International Edition*, *49*(5), 946-949.

[9] Xu, C., Zhang, B., Wang, A. C., Zou, H., Liu, G., Ding, W., ... & Wang, Z. L. (2019). Contact-electrification between two identical materials: curvature effect. *ACS Nano*, *13*(2), 2034-2041.

[10] Wang, Z. L. (2021). From contact electrification to triboelectric nanogenerators. *Reports on Progress in Physics*, *84*(9), 096502.

[11] Díaz-Ballester, A., Castillo-Anguera, S., Rafi, J. M., Gómez-Martínez, R., Abadal, G., Figueras, E., ... & Cañadas, J. C. (2014). Charge storage and retention in electret dielectric layers for energy harvesting applications. In *2014 IEEE 9th IberoAmerican Congress on Sensors* (pp. 1-4). IEEE.

[12] Chen G, Xu Z. Charge trapping and detrapping in polymeric materials. *Journal of Applied Physics*, ***106***, 123707 (2009).
