# Supplementary material for: Visualization and standardized quantification of surface charge density for triboelectric materials
Source: Nat Commun. 2024 Jul 17;15:6004. doi: 10.1038/s41467-024-49660-9 (PMC11255240; doi:10.1038/s41467-024-49660-9)
Supplement: Supplementary file 4 — Description of Additional Supplementary Files [file 41467_2024_49660_MOESM4_ESM.pdf]

Description of Additional Supplementary Files

File Name: Supplementary Movie 1

Description: Surface potential scanning of triboelectric materials.

File Name: Supplementary Movie 2

Description: Contactless response of posi-PTFE based TENG.

File Name: Supplementary Movie 3

Description: Contactless response of manipulator's finger towards a cube.

File Name: Supplementary Movie 4

Description: Contactless response of manipulator's finger towards a triangular prism with different shapes.
